# The arrhythmogenic cardiotoxicity of the quinoline and structurally related antimalarial drugs: a systematic review

**DOI:** 10.1186/s12916-018-1188-2

**Published:** 2018-11-07

**Authors:** Ilsa L. Haeusler, Xin Hui S. Chan, Philippe J. Guérin, Nicholas J. White

**Affiliations:** 1 WorldWide Antimalarial Resistance Network (WWARN), Oxford, UK; 2 Centre for Tropical Medicine and Global Health, Nuffield Department of Medicine, University of Oxford, Oxford, UK; 3 Mahidol-Oxford Tropical Medicine Research Unit (MORU), Faculty of Tropical Medicine, Mahidol University, Bangkok, Thailand; 4 Oxford University Hospitals NHS Foundation Trust, Oxford, UK

**Keywords:** Systematic review, Malaria, Antimalarials, Quinolines, Piperaquine, Electrocardiogram, QT, Arrhythmia, Torsade de pointes, Mass drug administration

## Abstract

**Background:**

Several quinoline and structurally related antimalarial drugs are associated with cardiovascular side effects, particularly hypotension and electrocardiographic QT interval prolongation. A prolonged QT interval is a sensitive but not specific risk marker for the development of Torsade de Pointes—a potentially lethal polymorphic ventricular tachyarrhythmia. The increasing use of quinoline and structurally related antimalarials in mass treatments to eliminate malaria rapidly highlights the need to review their cardiovascular safety profiles.

**Methods:**

The primary objective of this systematic review was to describe the documented clinical and electrocardiographic cardiovascular side effects of quinine, mefloquine, lumefantrine, piperaquine, halofantrine, chloroquine, sulfadoxine-pyrimethamine, amodiaquine, and primaquine. Trials in healthy subjects or patients with *Plasmodium falciparum* or *P. vivax* infection were included if at least two ECGs were conducted during the trial. All trial designs were included except case reports and pooled analyses. Secondary outcomes were the methods adopted by trials for measuring and reporting the QT interval.

**Results:**

Data from trials published between 1982 and July 2016 were included. A total of 177 trials met the inclusion criteria. 35,448 participants received quinoline antimalarials in these trials, of which 18,436 participants underwent ECG evaluation. Subjects with co-medication use or comorbidities including cardiovascular disease were excluded from the majority of trials. Dihydroartemisinin-piperaquine was the drug most studied (5083 participants). Despite enormous use over the past 60 years, only 1076, 452, and 150 patients had ECG recordings reported in studies of chloroquine, amodiaquine, and primaquine respectively. Transiently high concentrations of quinine, quinidine, and chloroquine following parenteral administration have all been associated with hypotension, but there were no documented reports of death or syncope attributable to a cardiovascular cause, nor of electrocardiographic recordings of ventricular arrhythmia in these trials. The large volume of missing outcome information and the heterogeneity of ECG interval reporting and measurement methodology did not allow pooled quantitative analysis of QT interval changes.

**Conclusions:**

No serious cardiac adverse effects were recorded in malaria clinical trials of 35,548 participants who received quinoline and structurally related antimalarials with close follow-up including 18,436 individuals who underwent ECG evaluation. While these findings provide further evidence of the rarity of serious cardiovascular events after treatment with these drugs, they also underscore the need for continued strengthening of pharmacovigilance systems for robust detection of rare drug adverse events in real-world populations. A standardised approach to measurement and reporting of ECG data in malaria trials is also needed.

**Trial registration:**

PROSPERO CRD42016036678

**Electronic supplementary material:**

The online version of this article (10.1186/s12916-018-1188-2) contains supplementary material, which is available to authorized users.

## Background

Quinoline antimalarials and structurally related compounds have long been known to cause cardiovascular side effects. Many of these antimalarial drugs cause hypotension, partly through alpha blockade, and they affect both depolarisation and repolarisation of cardiac and skeletal muscle [1]. The dangers of rapid intravenous injection of quinine, the marked prolongation of the electrocardiographic (ECG) QT interval caused by quinidine, and the lethality of chloroquine in overdose have each caused considerable concern over their use in the treatment of malaria. The discovery in 1993, after its registration, that halofantrine was associated with marked QT prolongation and sudden death augmented these concerns [2]. More recently, there has been uncertainty over the potential risks associated with the QT prolongation following use of piperaquine and the fixed-dose combination dihydroartemisinin-piperaquine (DP), the latest addition to the artemisinin-based combination therapies (ACTs) recommended for treatment of malaria by the World Health Organization (WHO) [3]. While the European Medicines Agency has approved DP, it called for more data to substantiate the cardiac safety of DP and its effect on the QT interval, particularly in children [4]. Consideration of this well-tolerated antimalarial drug for use in intermittent preventative therapy (IPT) and in mass drug administration (MDA) as part of malaria control interventions underlines the urgent need to clarify the cardiovascular safety profile of DP and structurally related antimalarials [5].

Characterising the electrophysiological effects of a drug, particularly during the treatment of acute illness, is not straightforward. It is not a problem unique to antimalarials and is a major consideration during drug development. A prolonged QT interval, reflecting a delay in ventricular repolarisation, is a sensitive but not specific risk marker for ventricular tachyarrhythmia development, notably Torsade de Pointes (TdP) [6]. This rhythm if sustained can degenerate into ventricular fibrillation and result in sudden cardiac death. The relationship between QT prolongation and the risk of developing ventricular tachyarrhythmias is incompletely understood, although many factors are known to contribute. These include the presence of underlying genetically determined QT prolongation, electrolyte abnormalities, structural heart disease, female gender, and co-administration with other drugs which also prolong the QT interval or increase drug levels [6]. Without detailed investigation, it is therefore difficult to assess an individual’s risk of a drug precipitating life-threatening ventricular tachyarrhythmias.

Acute malaria illness itself has significant effects on the QT interval [1]. Prior to treatment, patients are usually febrile, anxious, tachycardic, and often anorexic and nauseated. The sympathetic nervous system is activated, and the QT interval shortens. As the patient recovers, often supine in bed, the fever settles, appetite returns, and the heart rate declines. The QT interval lengthens. The difference between the pre-treatment shortened interval and the third day normalised value (which often coincides with peak antimalarial drug concentrations) is often misattributed to a drug effect [1]. If there is a drug effect, then it may be compounded by this systemic physiological response to recovery.

The only previous systematic review on antimalarial cardiotoxicity analysed case reports of deaths secondary to possible halofantrine-related cardiotoxicity [7]. To clarify the cardiovascular safety profile of DP and structurally related antimalarials, we assessed, by systematic review of published clinical trials, the frequency of reported clinical and electrocardiographic cardiac adverse effects after use of quinoline and structurally related antimalarials for malaria-related indications, with a focus on QT interval prolongation.

## Methods

### Eligibility criteria

The nine antimalarial drugs included in this review were quinine, mefloquine, lumefantrine, piperaquine, halofantrine, chloroquine, sulfadoxine-pyrimethamine (SP), amodiaquine, and primaquine. SP, a non-quinoline containing combination antimalarial, was included in the review as a negative control as it has negligible effects on the QT interval. Trials which administered these drugs as antimalarials to patients with *Plasmodium falciparum* or *P. vivax* mono- or mixed infections or to healthy participants, and in which at least two systematic electrocardiograms (ECGs) were performed, were eligible for inclusion. All trial designs except case reports and pooled analyses were included. Conference abstracts were excluded. All ages and populations, including children and pregnant women, were included. There was no restriction of date of publication or language of article. This review conformed to the PRISMA statement and has been registered in the PROSPERO database (CRD42016036678).

### Search strategy and study selection

Articles were identified between October 2015 and July 2016 through electronic searches of the MEDLINE, Embase, and Global Health databases (Additional file 1) and by using reference lists of relevant articles. Records were deduplicated using Mendeley (Mendeley Ltd., Version 1.17.8). Abstracts were reviewed to assess eligibility; the full article was obtained for assessment if there was doubt as to the relevance of an article. Unpublished literature was not included, and abstracts were excluded if the full-text article could not be obtained.

### Data extraction and review outcomes

Data were extracted into a structured database. Variables included study year and location, trial design, population demographics, drug(s) assessed, ECG measurement methodology and timepoints, drug measurement and pharmacokinetic analysis methodology, and clinical and electrocardiographic cardiovascular adverse events (Additional file 2). Patient series were compared with those of other included articles to minimise data duplication. Primary outcomes were the number and character of clinical cardiovascular adverse events (palpitations, syncope, or sudden cardiac death) and ECG-documented arrhythmias (including ventricular tachycardia, ventricular fibrillation, and TdP). Additional primary outcomes included the mean QT interval corrected with Bazett’s formula at baseline, 4 and 24 h, and 7 days after drug administration and the proportion of patients who developed a prolonged QT interval during the trials. Secondary outcomes were the features of ECG methodology and QT interval analysis adopted (Additional file 3).

### Risk of bias

A standard set of criteria were used to assess bias in each study (Additional file 4). This included assessment of blinding of participants, study personnel, and ECG readers; selective outcome reporting and completeness of outcome data; and method of electrocardiographic interval measurement. The proportion of studies with a low, unclear, or high risk of bias for each criterion was calculated per drug.

## Results

**Fig. 1:**
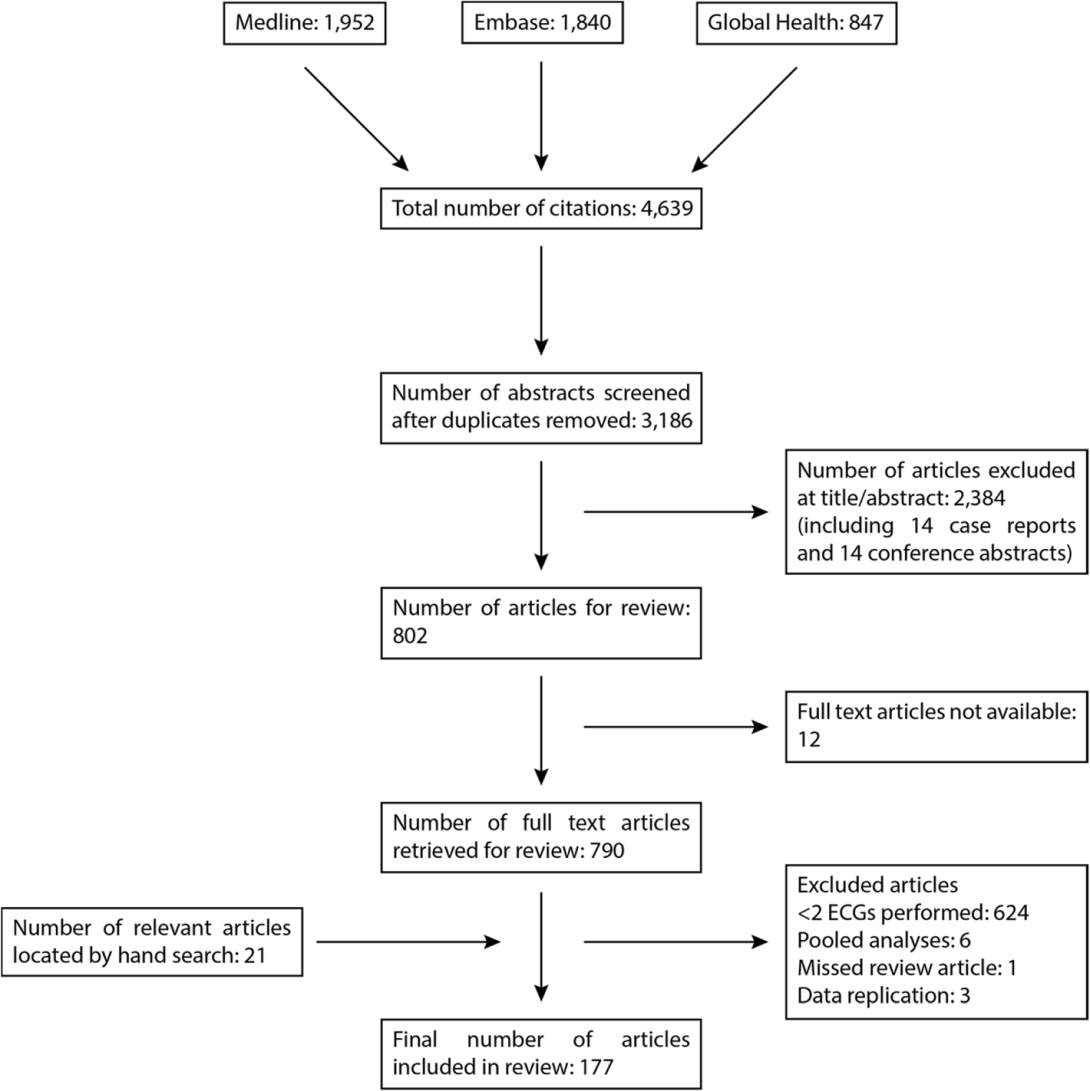
Flow diagram of study selection

In total, 177 articles (Fig. 1, Additional file 5) enrolling a total of 39,960 participants were included in the review. Of these participants, 35,448 received at least one of the drugs relevant to this review and 18,436 underwent ECG evaluation (Table 1).

**Table 1:** Total number of studies and participants per antimalarial drug, with number of studies including children and pregnant women

Drug	Total number of studies	Total number of participants who had drug	Total number of participants who had ECGs	Total number of studies which included children	Total number of studies which included pregnant women	Total number of studies which included pharmacokinetic analysis
Quinine	51	2611	2320	18	1	19
Mefloquine	45	7874	3099	13	2	19
Lumefantrine	39	5703	4787	19	3	19
Piperaquine	24	15,224	5083	7	1	12
Halofantrine	22	822	774	7	0	10
Chloroquine	17	1207	1076	6	0	10
Sulfadoxine-pyrimethamine	14	1288	695	4	2	5
Amodiaquine	10	569	452	2	1	8
Primaquine	7	150	150	0	0	4

Forty-nine studies evaluated more than one of the drugs included in this review. These articles have been included under each of the appropriate drugs for analysis

Quinine, mefloquine, and lumefantrine were the most intensively studied drugs by number of published studies (51, 45, and 39 studies respectively), whereas piperaquine and lumefantrine had the largest number of participants undergoing ECG investigation (5083 and 4787 participants respectively). Despite vast use of chloroquine over the last six decades (hundreds of tonnes consumed annually), only 1076 participants in 17 studies of chloroquine underwent ECG investigation. Amodiaquine and primaquine have been the least intensively investigated (10 and 7 studies respectively) with 452 and 150 participants respectively undergoing ECG investigation. Fifty-nine studies included children, most of which were trials of quinine, mefloquine, or lumefantrine. Seven trials enrolled pregnant women (1232 participants).

### Study characteristics

Overall, articles were published between 1982 and 2016 (Table 2).

**Table 2:** Summary of study characteristics for each antimalarial drug

	Quinine (51 studies)	Mefloquine (45 studies)	Lumefantrine (39 studies)	Piperaquine (24 studies)	Halofantrine (22 studies)	Chloroquine (17 studies)	SP (14 studies)	Amodiaquine (10 studies)	Primaquine (7 studies)
Range of dates studies were published	1982–2014	1983–2012	1997–2014	2003–2016	1993–2004	1983–2014	1983–2015	1987–2014	1992–2015
Countries where studies were most frequently conducted in (% of studies)	Thailand (43%) Vietnam (8%) Myanmar (8%)	Thailand (54%) Brazil (11%)	Thailand (23%) Kenya (13%)	Thailand (25%) Cambodia (17%)	France (32%) Nigeria (18%)	Thailand (41%) India (24%)	Brazil (36%) Thailand (21%)	No majority (each trial in different country)	Thailand (71%)
Median (range) number of participants	59 (7–561)	94 (8–3673)	165 (12–1553)	130 (12–10,925)	38.5 (8–120)	58 (11–456)	99 (20–3673)	32 (10–336)	16 (8–329)
% (number) of studies OLRCT	43 (22)	56 (25)	36 (14)	54 (13)	18 (4)	18 (3)	50 (7)	40 (4)	14 (1)
% (number) of studies DBRCT	6 (3)	24 (11)	18 (7)	8 (2)	9 (2)	35 (6)	36 (5)	0 (0)	14 (1)
% (number) of studies OLRCTX	0 (0)	2 (1)	10 (4)	8 (2)	14 (3)	6 (1)	0 (0)	40 (4)	57 (4)
% (number) of studies non-comparative	29 (15)	9 (5)	21 (8)	13 (3)	45 (10)	6 (1)	0 (0)	10 (1)	14 (1)
% (number) of studies primary outcome CV safety	20 (10)	13 (6)	13 (5)	13 (3)	41 (9)	18 (3)	14 (2)	20 (2)	0 (0)
% (number) of trials PK or PK/PD primary aim	26 (13)	24 (11)	26 (10)	21 (5)	27 (6)	53 (9)	7 (1)	60 (6)	71 (5)
% (number) of papers PK/PD primary aim	6 (3)	9 (4)	10 (4)	4 (1)	13 (3)	18 (3)	0 (0)	10 (1)	0 (0)

*SP* sulfadoxine-pyrimethamine, *OLRCT* open-label randomised control trial, *DBRCT* double-blind randomised control trial, *OLRCTX* open-label randomised control crossover trial, *CV* cardiovascular, *PK* pharmacokinetic, *PD* pharmacodynamic

Most quinine, mefloquine, piperaquine, chloroquine, and primaquine trials were conducted in Thailand, and the majority of halofantrine trials were reported from France. Although African countries are underrepresented (Additional file 6), the majority of lumefantrine and amodiaquine trials were conducted in countries throughout sub-Saharan Africa.

Sixty-eight of the 177 trials (38%) were open-label randomised control trials, 41/177 (23%) were non-comparative trials, and 26/177 (15%) were double-blind randomised control studies. Only 29/177 trials (16%) specified that their primary aim was to investigate cardiovascular safety, and 15/177 trials (8%) were designed to investigate pharmacokinetic-pharmacodynamic relationships. For halofantrine, 9/22 trials (41%) had investigation of cardiovascular safety as the primary aim, with only 3/24 (13%) for piperaquine.

The total number of participants included in each trial ranged from 7 to 10,925. Lumefantrine trials had the largest median number of participants per trial (*n* = 165) followed by piperaquine (*n* = 130), and the fewest participants were in primaquine trials (*n* = 16).

### Patient characteristics

Trial populations were young with mean ages in the 20s (range 19.9 [halofantrine] to 33 years [primaquine]), although there was a large proportion of missing data (Table 3). The numbers of male and female participants were not reported in 27/177 (15%) of trials. In the other trials, there were a total of 14,921 males and 8502 females.

**Table 3:** Summary of patient characteristics for each antimalarial drug

	Quinine (51 studies)	Mefloquine (45 studies)	Lumefantrine (39 studies)	Piperaquine (24 studies)	Halofantrine (22 studies)	Chloroquine (17 studies)	SP (14 studies)	Amodiaquine (10 studies)	Primaquine (7 studies)
Median of mean age (years)	25.6	26	26	23.4	19.9	20.8	–	22.1	33
% missing data for mean age	55	80	64	38	41	71	93	60	57
Median of median age (years)	16.4	24	25	23	23.8	–	–	–	–
% missing data for median age	93	96	85	88	91	94	100	100	100
Age range (years)	0.3–90	0.4–88	0.2–75	6–65	0.25–84	1–74	12–62	0.8–65	16–74
Total number of males	2571	5137	5226	4522	684	1052	723	287	373
Total number of females	1141	1848	4233	2682	256	422	377	519	118
% (number) trials including healthy participants	14 (7)	22 (10)	28 (11)	29 (7)	18 (4)	35 (6)	14 (2)	40 (4)	71 (5)
% (number) trials including P. falciparum infection	88 (45)	78 (35)	72 (28)	71 (17)	77 (17)	41 (7)	86 (12)	60	14 (1)
% (number) trials including P. vivax infection	4 (2)	0 (0)	5 (2)	17 (4)	14 (3)	29 (5)	7 (1)	0 (0)	14 (1)
% (number) trials including uncomplicated malariainfection	45 (23)	69 (31)	72 (28)	71 (17)	77 (17)	35 (6)	71 (10)	60 (6)	29 (2)
% (number) trials including complicated or severe malaria infection	51 (26)	4 (2) (both studies also trialled quinine)	0 (0)	0 (0)	0 (0)	12 (2) (1 study just chloroquine, the other also trialled quinine)	0 (0)	0 (0)	0 (0)
% (number) trials excluding any medical comorbidities	31 (16)	58 (26)	62 (24)	71 (17)	68 (15)	65 (11)	57 (8)	90 (9)	86 (6)
% (number) trials specifically excluding cardiovascular comorbidities	12 (6)	16 (7)	49 (19)	38 (9)	46 (10)	41 (7)	14 (2)	60 (6)	57 (4)
% (number) trials excluding any co-medication use	65 (33)	53 (24)	62 (24)	42 (10)	54 (12)	71 (12)	57 (8)	100 (10)	86 (6)
% (number) trials excluding co-medication with antimalarials	57 (29)	44 (20)	39 (15)	13 (3)	36 (8)	24 (4)	50 (7)	80 (8)	43 (3)
% (number) trials excluding drugs which interfere with cardiovascular system	6 (3)	2 (1)	23 (9)	13 (3)	18 (4)	12 (2)	0 (0)	30 (3)	0 (0)

Unavailable data is indicated by ‘–’

*SP* sulfadoxine-pyrimethamine

#### Malaria patients

Seventy-two percent (127/177) of trials included participants with *P. falciparum* mono- or mixed infection. Sixteen trials included patients with *P. vivax* infection specifically, and only 4 of these investigated participants with *P. vivax* mono-infections (3 trialled chloroquine and 1 trialled halofantrine) [8–11]. The majority of studies were of patients with uncomplicated malaria (106/177, 60%), and 15% were of severe or complicated malaria (27/177).

#### Healthy subjects

Forty-seven of the 177 trials (27%) included healthy participants. Such trials were generally pharmacokinetic studies or trials of intermittent preventative therapy.

#### Exclusion criteria

Overall, 34/177 studies (19%) did not detail any exclusion criteria. In 103/177 studies (58%), participants with at least one medical comorbidity were excluded. In the remainder of trials, it was not specifically mentioned if medical comorbidities were excluded. In 29% of trials (52/177), cardiovascular comorbidities were stated to have been excluded, with only three trials specifically detailing that cardiovascular comorbidities were not excluded. Similarly, in 63% (112/177), participants who had co-medications (most commonly other antimalarials and other drugs which interfere with the cardiovascular system) were excluded and only one trial did not exclude participants with co-medications. Seventy-nine of the 177 trials (45%) excluded participants with both comorbidities and co-medication use, and there were no trials which included both groups of participants.

### Primary outcomes

There were no reports of death attributable to a cardiovascular cause in any trial included in this review. There were no electrocardiographic recordings of ventricular tachycardia, ventricular fibrillation, or TdP. Other ECG abnormalities were described, the most common of these being bradycardia and first-degree atrioventricular block (Additional file 7).

#### Bradycardia

Bradycardia was reported in 3.9% of 3099 participants after mefloquine [2, 12–16], 1.6% of 774 after halofantrine [2, 17], and 10.8% of 452 after amodiaquine [18, 19]. Although nausea and vomiting are common in malaria, particularly in children, and may be provoked by antimalarial drugs, the association with nausea was not reported.

#### Atrioventricular block

Halofantrine was associated with atrioventricular block: 25 episodes of first-degree block were reported of 774 participants who had ECGs (3.2%) [2, 17, 20], while two children had second-degree Mobitz type 1 (Wenkebach) block after treatment for falciparum malaria [2, 21]. Following quinine, amodiaquine, and mefloquine (with SP), 2.5% (57/2320), 0.4% (2/452), and 0.2% (7/3099) respectively of participants with ECG recordings developed first-degree heart block [13, 15, 22–24].

#### Others

Other ECG abnormalities were described in patients undergoing treatment for cerebral malaria although investigators attributed these to severity of malaria illness rather than drug treatment [12, 25, 26]. A 20-year-old male developed Wolff-Parkinson-White syndrome following piperaquine which was not detected on baseline ECGs [27]. The significance of this apparent revelation of an accessory conduction pathway is uncertain.

#### QT assessment

Due to the heterogeneity of ECG methodology, analytical techniques and reporting, as well as the large volume of missing reported information, it was not possible to perform the planned analysis to quantitate the antimalarial drug effects on the QT interval. The mean maximal change from baseline (Bazett’s corrected QT interval, QTcB) and the individual maximum QTcB or QTcF (Fridericia’s corrected) values were the most frequently detailed outcomes but were insufficient to perform a quantitative analysis.

Analytical and reporting heterogeneity included using different measures of central tendency (mean or median), reporting the QT interval corrected for heart rate variation by different methods, reporting absolute values at different time points (as ECGs were performed at various time points across studies), or changes from a certain time (most often the baseline) either as an absolute value or a proportion. Many studies, despite performing ECGs as part of the trial, either did not report ECG findings or only stated that none were clinically relevant.

### Secondary outcomes

Additional file 8 shows the summary of secondary outcomes for each drug.

#### Assessments of the temporal relationship of ECG changes to drug concentrations

In total, 87 studies included pharmacokinetic data. Quinine, mefloquine, and lumefantrine have undergone the most PK evaluations, each with 19 trials measuring drug concentrations. Overall, 74 of 177 trials (41%) specified food intake of participants around the time of drug dosing.

Apart from baseline ECGs taken before administration of the first dose of drug, the overwhelming majority of trials did not specify whether subsequent ECGs were taken before drug administration (likely to be trough level of drug) or following drug administration (higher drug levels, which could include peak drug levels). Only one trial of primaquine specified that all ECGs were taken in the morning to minimise the effects of diurnal variation of the QT interval [28].

#### QT interval measurement method

Overall, 118 of 177 trials (67%) did not specify the method of ECG reading (manual or automatic). Nineteen percent (*n* = 34) of trials reported manually reading ECGs, 3% (*n* = 6) used only automatic readings, and 11% (*n* = 19) used both. van Vugt and colleagues included both manual and automatic measurements and found automatically measured values were generally higher than manually measured values, and this difference was greater at larger QTc values [29]. A piperaquine trial was stopped prematurely on the basis of electronic measurements of the QTc because the machine measured the QU interval as the QT, despite correct manual readings on the same traces [30]. Some more recent trials used electronic rulers for manually reading the QT interval [30–32]. In total, there were five trials which sent their ECGs to a centralised ECG laboratory for assessment, each of which investigated piperaquine [32–36].

#### QT rate correction

The majority of trials did not specify which QT correction formula was used, although every trial reporting QT intervals reported corrected QT values. Fifteen trials used two or more correction formulae. Twenty-seven percent (*n* = 48) of trials used Bazett’s formula alone. Bazett’s and Fridericia’s were both reported in 10% (*n* = 12) of trials. Five trials [18, 31, 37–39] used alternative formulae, including Wernicke et al.’s [40], Hodges’ and Karen [41].

#### Criteria for QT prolongation

There were many definitions used (Additional file 9), specified in only 64 of 177 studies (36%). Definitions included absolute values with thresholds between 420 and 470 ms, with many trials using different values for men and women, such as > 430 ms (males) and > 450 ms (female). Other definitions included a proportional increase, commonly 25% increase from baseline, or an absolute increase, such as > 30 or > 60 ms from baseline. Most trials used the QT corrected using Bazett’s formula for these definitions.

### Risk of bias

Additional file 10 shows the assessment of methodological quality of individual studies, and Additional file 11 summarises the risk of bias for each criterion per drug. In the majority of trials, neither the participants nor the investigators (especially ECG readers) were blinded to treatment allocation. Most trials did not specify cardiovascular or ECG outcomes. Many studies were limited by incomplete reporting of ECG methodology.

## Discussion

This systematic review assessed available published prospective trials to determine the incidence and severity of clinical and electrocardiographic cardiovascular adverse effects of antimalarial drugs. The primary focus was QT prolongation and related arrhythmic cardiotoxicity after use of the quinoline and structurally related antimalarials for malaria. There were no sudden deaths attributed to cardiac arrhythmias recorded in the > 35,000 individuals who received the quinoline and structurally related antimalarials in the 177 clinical trials included in this review. Among the > 18,000 subjects who underwent ECG evaluation, a variety of generally non-serious self-limiting cardiac rhythm abnormalities were described usually without contextual information, making interpretation of causation difficult. Balanced against the clear life-saving benefits of giving effective antimalarials promptly in malaria, with the exception of halofantrine, concerns over cardiotoxicity have not limited the current use of the quinoline and structurally related antimalarial drugs.

These findings provide further evidence of the rarity of serious cardiovascular events after treatment with the quinoline and structurally related antimalarials, although the precise estimation of risk is limited, because of this rarity, by the total size of the source data available despite an inclusive search strategy. In this review, the median number of participants per trial ranged from 16 to 165 among the nine antimalarials studied. Such individual study sample sizes are designed to evaluate drug efficacy and are too small, even when pooled, to characterise the risk of very rare (< 1/10,000) drug adverse events such as Torsade de Pointes [42, 43]. The representativeness of the clinical trial population of potential recipients of population-based drug administration, e.g. in terms of age, gender, ethnicity, and cardiovascular risk factors, is another potential limitation. Fifty-eight percent (103/177) and 63% (112/177) of included studies listed medical comorbidities and co-medications as exclusion criteria, while healthy volunteer studies, often of adult males from non-malaria endemic countries, comprised 27% (47/177) of the included studies.

The importance of robust detection and evaluation of extremely rare and serious adverse events such as sudden unexplained death in real-world populations and the implications of such findings for population-based drug administration strategies underscore the need for ongoing synthesis of all available clinical evidence. Post-marketing pharmacovigilance approaches such as spontaneous individual case safety reporting are especially important in signal detection of very rare adverse events despite challenges in assessing causality [43]. For example, the two sentinel cases of sudden death and collapse with extreme QTc interval prolongation after halofantrine given for the treatment of clinical malaria were important in stimulating the accumulation of further evidence which confirmed the arrhythmogenic effects of the drug [2, 7]. The findings of this review should therefore be interpreted in the context of this wider evidence base and the intended treatment indication(s) for each antimalarial.

The quinoline antimalarials have antiarrhythmic effects, best illustrated by quinidine, the d-diastereomer of quinine. Quinidine has been used mainly as an antiarrhythmic and it can cause TdP. It produces substantially greater QT prolongation than quinine. Quinidine and quinine are now used mostly in the treatment of severe falciparum malaria which itself has a significant risk of mortality. The benefit of effective treatment of malaria where these are the only parenteral drugs available outweighs the potential risks of cardiotoxicity [3].

Chloroquine is the most widely used antimalarial drug in history. It has a terminal elimination half-life of one month and an annual consumption of hundreds of tonnes for over 50 years, so it may be the drug to which humans have been exposed to most [1]. Despite producing consistent QT prolongation, the only case reports of TdP and sudden death have been for its use for non-malaria indications such as systemic lupus erythematosus or rheumatoid arthritis, where high doses are used for much longer than in malaria treatment, or in overdose [44].

Halofantrine is the only antimalarial drug considered to have an unacceptable arrhythmogenic risk when used for malaria indications [44]. The earliest report of its cardiotoxicity provided evidence of both extreme QT prolongation, conduction delay, and clinical cardiovascular adverse effects [2]. A 2009 review of the published literature and the GlaxoSmithKline Global Safety Database found 35 cases of fatal cardiotoxicity after halofantrine use between 1988 and 2005 [7]. Of the 35 cases, 26 had one or more risk factors for cardiotoxicity, including underlying cardiovascular disease or other comorbidities, concomitant use of a drug which can cause QT prolongation, administration with food, and higher than recommended doses given. As for other drugs, females were at greater risk; 70% of the patients who died were female. In all five paediatric deaths, either there was a contraindication to halofantrine use, or a higher dose was given in error.

DP is the most recent ACT to be recommended by WHO as first-line treatment of malaria. Its registration coincided with increased regulatory scrutiny of drugs which prolong the QT interval (most of the older antimalarials were introduced before awareness of the arrhythmogenic risk associated with this effect). This review identified 24 trials of piperaquine with systematic ECG assessment, with 7 trials including children and one including pregnant women. Of the 8 trials which included healthy participants, 2 trials investigated DP for use as IPT, 5 were PK trials in healthy participants, and 1 was a PK trial in healthy participants and participants infected with *P. falciparum*. In these 24 studies, there were no reports of sudden death suggestive of a fatal arrhythmia nor of any other major cardiovascular adverse outcomes following piperaquine use. A systematic review and meta-analysis was recently published (after the period of this review) of nearly 200,000 DP-treated individuals, including over 150,000 individuals in unpublished studies of mass drug administration in which exclusion of TdP risk factors was not possible [45]. The review reported one case of sudden death following DP use in MDA of a previously healthy 16-year-old female in Mozambique who developed palpitations after her second dose of DP, then collapsed, and died on the way to hospital (no ECG or autopsy was performed). This case of sudden unexplained death was considered possibly drug-related by cardiology and pharmacology experts at the WHO Evidence Review Group on the Cardiotoxicity of Antimalarials (there are also non-drug-related and non-cardiogenic causes of sudden unexplained death) [44]. The subsequent meta-analysis found that the risk of sudden unexplained death within 30 days of taking DP was no higher than the baseline risk of sudden cardiac death over the same period [45]. In addition, despite millions of doses having been distributed, there have been no cases of TdP after DP reported to global pharmacovigilance databases [44].

Pyronaridine is structurally related to the quinoline antimalarials, and its effect on the QT interval has been investigated in clinical trials of antimalarial efficacy. Artesunate-pyronaridine is a highly effective antimalarial drug which has been studied in > 3500 individuals in both pre- and post-registration studies [9, 16, 28, 46–49]. The most extensive of these studies has recently been reported after the period of this review [46]. This included a total trial population of nearly 5000 people, of whom 1342 received artesunate-pyronaridine. Of the other trials, all but one included other antimalarials included in this review [48]. There have been no marked ECG changes and no cardiovascular adverse effects attributed to artesunate-pyronaridine reported in any of these trials.

The QT interval is the most frequently used clinical biomarker for assessing the potential for the development of ventricular tachyarrhythmias and thus risk of sudden cardiac death. However, it is a surrogate marker, which while sensitive, has limited specificity. Its interpretation is further compromised by the extensive heterogeneity of the methods used in its measurement and reporting. Also, many factors affect the QT interval. These include patient factors such as age, gender, genetic predisposition, and comorbidities such as myocardial ischaemia and electrolyte disturbances [6]. The time of day the recording is made (the effects of circadian rhythm), position of the patient, food intake (independent of the effect of food on drug pharmacokinetics), and drug-drug interactions (with drugs which prolong the QT or increase drug concentrations including traditional medicines) also affect the QT interval. These variables are very difficult to control, particularly in the context of clinical trials involving patients with acute malaria infection.

Acute malaria infection is associated with disease factors such as fever, sympathetic activation, and tachycardia which can affect the QT interval; they are therefore confounders. These effects of malaria (particularly recovery from malaria) and other covariates and their effect on the QT interval have not been characterised adequately. Studying healthy controls allows a pure assessment of drug effects but does not allow characterisation of a disease-drug interaction. Comparing pre-dose versus post-dose ECGs which are recorded at the same times as plasma concentration measurements reduces the effects of these confounders, but there are relatively few such data. More comprehensive reporting of food intake and time of ECG measurement relative to drug intake as well as characterisation of antimalarial drug absorption profiles would improve assessments in future studies, with peak rather than trough drug levels being of greater relevance in the evaluation of potential cardiovascular toxicity.

This work has highlighted important methodological issues which confounded a standardised assessment of the cardiovascular effects of the quinoline and structurally related antimalarial drugs. There is considerable variation in the recording and measurement of the QT interval in antimalarial drug trials. The QT interval is technically difficult to measure, even by experts, and the process of measurement introduces further confounding factors and systematic error [50]. There is also substantial variation in the interpretation and reporting of the QT interval in a clinical context.

Automated measurements are considered not as accurate or reliable compared with manual measurements [51]. Many machines have problems with identifying U waves which can be mistaken for the T wave: a recent trial assessing high-dose DP was stopped because of apparent QT prolongation when the machine read the QU interval as the QT interval [30]. T and U wave morphology can be difficult to identify even manually, and readings are subject to a high degree of inter-user variability [50]. For paper recordings, a slower speed of recording, at 25 mm/s rather than 50 mm/s, makes the interval more difficult to measure if manual readings are employed. Some recent studies have used computer-aided ECG interpretation by specialist personnel at centralised laboratories. Whilst some investigators take multiple QT interval measurements across different leads and calculate an average, there is no agreement over this method. There are also many definitions of the end of the T wave used by trials.

Another source of potential confusion is the heart rate correction. This is necessary because the QT interval has an inverse relationship with heart rate. Several correction formulae are used, but the choice of the best formula is the subject of ongoing debate. The most widely used are Bazett’s and Fridericia’s formulae. In a healthy population, Bazett’s formula overestimates at higher heart rates (lengthens the QTc interval) and underestimates at lower heart rates (shortens the QTc interval). In healthy subjects, Fridericia’s provides better, although still imperfect correction at heart rates < 60 and > 100 beats per minute [6]. Where possible, the QT correction formula used should be derived for the study population, but this requires a large study and sufficient variation in heart rates [52].

Interpretation of the QTc interval in a clinical context is difficult because different populations have different normal QTc ranges. There are broadly accepted ‘normal’ QTc values, but many different values of ‘abnormally long’ exist and are used, ranging from > 420 to ≥ 500 ms in this review. This reflects uncertainty over the relationship between degree of prolongation and risk of arrhythmia development although a QT/QTc interval of > 500 ms is generally accepted to be a threshold for clinical concern [53].

More specific alternatives to evaluation of QT/QTc prolongation for determination of drug TdP risk are being developed, including through the Comprehensive in vitro Proarrhythmia Assay (CiPA) initiative, a multi-stakeholder global effort among regulators, industry, and academia. CiPA proposes a mechanistic-based, four-component approach coupling in vitro assessment of drug effects on multiple ion currents with an in silico computational model of the human ventricular cardiomyocyte for predicting proarrhythmic risk. These assessments would be followed by confirmatory in vitro studies on human stem cell-derived cardiomyocytes and in vivo phase I ECG safety evaluation. A validation programme is ongoing, and if successful and adopted by regulators, a CiPA evaluation demonstrating low arrhythmogenic risk could potentially obviate the need for intensive ECG monitoring in clinical phase III trials of QT/QTc prolonging drugs.

## Conclusions

Several of the quinoline and structurally related antimalarial drugs are associated with electrocardiographic QT prolongation, but the only drug clearly associated with harm when used for the treatment of malaria is halofantrine. There have been no reports of death or syncope attributable to a cardiovascular cause nor electrocardiographic traces recording ventricular arrhythmia captured during malaria clinical therapeutic trials of other quinoline or structurally related antimalarial drugs which included systematic ECG assessment. While these findings add to existing evidence from individual case report databases in supporting the rarity of these adverse events, they also underscore the need for continued strengthening of pharmacovigilance systems for robust detection of such rare drug adverse events in real-world populations. The variable definitions, procedures, and analytical methods employed precluded systematic analysis of the QT interval. Pooled analyses of individual patient clinical trial data including from IPT and MDA studies are important next steps to determine the effect of the quinoline antimalarials on the QT interval. A standardised approach to measurement and reporting of ECG data in malaria trials is also urgently needed.

## Additional files

Additional file 1: Search strategies from Ovid MEDLINE, Embase, and Global Health. (DOCX 25 kb)

Additional file 2: Complete list of variables extracted from articles. (DOCX 15 kb)

Additional file 3: List of primary and secondary outcomes. (DOCX 14 kb)

Additional file 4: Criteria used for assessment of risk of bias of individual studies. (DOCX 17 kb)

Additional file 5: List of references included in the review. (DOCX 28 kb)

Additional file 6: Number of studies conducted in each region, for each drug. (DOCX 14 kb)

Additional file 7: Other cardiovascular events observed, organised by arrhythmia. (DOCX 17 kb)

Additional file 8: Summary of secondary outcomes, for each drug. (DOCX 17 kb)

Additional file 9: List of definitions of prolongation used per drug. (DOCX 18 kb)

Additional file 10: Assessment of risk of bias for individual studies. (DOCX 26 kb)

Additional file 11: Risk of bias for each criterion per drug. (PDF 621 kb)

## Abbreviations

ACTs: Artemisinin combination therapies
DP: Dihydroartemisinin-piperaquine
ECG(s): Electrocardiogram(s)
ICH: International Conference on Harmonisation of Technical Requirements for Registration of Pharmaceuticals for Human Use
MORU: Mahidol-Oxford Tropical Medicine Research Unit
PD: Pharmacodynamic
PK: Pharmacokinetic
PRISMA: Preferred Reporting Items for Systematic Reviews and Meta-analyses
QTc: Heart rate-corrected QT interval
QTcB: Bazett’s formula-corrected QTc
QTcF: Fridericia’s formula-corrected QTc
SP: Sulfadoxine-pyrimethamine
TdP: Torsade de Pointes
WHO: World Health Organization
WWARN: WorldWide Antimalarial Resistance Network
