# Supplementary material for: The arrhythmogenic cardiotoxicity of the quinoline and structurally related antimalarial drugs: a systematic review
Source: BMC Med. 2018 Nov 7;16:200. doi: 10.1186/s12916-018-1188-2 (PMC6220451; doi:10.1186/s12916-018-1188-2)
Supplement: Supplementary file 1 — Search strategies from Ovid MEDLINE, Embase, and Global Health. (DOCX 25 kb) [file 12916_2018_1188_MOESM1_ESM.docx]

**Additional file 1** Search strategies from Ovid MEDLINE, Embase, and Global Health

**Ovid MEDLINE** (searched 22^nd^ October 2015)

1. Malaria/

2. Malaria, Cerebral/

3. Malaria, Falciparum/

4. Malaria, Vivax/

5. plasmodium falciparum/

6. plasmodium vivax/

7. malaria.ti,ab.

8. falciparum.ti,ab.

9. vivax.ti,ab.

10. plasmodium.ti,ab.

11. or/1-10

12. dihydroartemisinin-piperaquine.ti,ab.

13. (dihydroartemisinin adj piperaquine).ti,ab.

14. piperaquine.ti,ab.

15. chloroquine.ti,ab.

16. quinine.ti,ab.

17. amodiaquine.ti,ab.

18. artesunate-amodiaquine.ti,ab.

19. (artesunate adj amodiaquine).ti,ab.

20. lumefantrine.ti,ab.

21. benflumetol.ti,ab.

22. artemether-lumefantine.ti,ab.

23. artemether-benflumetol.ti,ab.

24. (artemether adj lumefantrine).ti,ab.

25. (artemether adj benflumetol).ti,ab.

26. coartem.ti,ab.

27. halofantrine.ti,ab.

28. mefloquine.ti,ab.

29. artesunate-mefloquine.ti,ab.

30. (artesunate adj mefloquine).ti,ab.

31. primaquine.ti,ab.

32. sulfadoxine.ti,ab.

33. pyrimethamine.ti,ab.

34. (sulfadoxine adj pyrimethamine).ti,ab.

35. (pyrimethamine adj sulfadoxine).ti,ab.

36. sulfadoxine-pyrimethamine.ti,ab.

37. pyrimethamine-sulfadoxine.ti,ab.

38. Amodiaquine/ad, ae, ct, pk, pd, po, to, tu, me, ur, bl, aa

39. Mefloquine/ad, ae, ct, pk, pd, po, to, tu, me, ur, bl, aa

40. Chloroquine/ad, ae, ct, pk, pd, po, to, tu, me, ur, bl, aa

41. Quinine/ad, ae, ct, pk, pd, po, to, tu, me, ur, bl, aa

42. Primaquine/ad, ae, ct, pk, pd, po, to, tu, me, ur, bl, aa

43. Sulfadoxine/ad, ae, ct, pk, pd, po, to, tu, me, ur, bl, aa

44. Pyrimethamine/ad, ae, ct, pk, pd, po, to, tu, me, ur, bl, aa

45. Drug Administration Schedule/

46. or/12-45

47. Electrocardiography/

48. Electrocardiography, Ambulatory/

49. Cardiotoxicity/

50. Arrhythmias, Cardiac/ci, co, di, pp

51. Heart Conduction System/ab, de, pp

52. Long QT Syndrome/ci

53. Torsades de Pointes/ci

54. Cardiovascular Diseases/ci, co

55. Heart/de

56. Heart rate/de, ph, pd

57. Blood Pressure/co, de, pd, ph, th

58. (QT OR QTc).ti,ab.

59. (QT adj (interval$ OR prolongation)).ti,ab.

60. (QTc adj (interval$ OR prolongation)).ti,ab.

61. (electrocardiogra$ OR ECG).ti,ab.

62. (cardiac adj toxicity).ti,ab.

63. cardiotoxicity.ti,ab.

64. toxic$.ti,ab.

65. safety.ti,ab.

66. (adverse adj effect$).ti,ab.

67. (blood adj pressure).ti,ab.

68. pharmacokinetic$.ti,ab.

69. or/47-68

70. 11 AND 46 AND 69

**Embase** (searched 22^nd^ October 2015)

1. malaria/

2. brain malaria/

3. malaria falciparum/

4. plasmodium vivax malaria/

5. plasmodium falciparum/

6. plasmodium vivax/

7. malaria.ti,ab.

8. falciparum.ti,ab.

9. vivax.ti,ab.

10. plasmodium.ti,ab.

11. or/1-10

12. dihydroartemisinin-piperaquine.ti,ab.

13. (dihydroartemisinin adj piperaquine).ti,ab.

14. piperaquine.ti,ab.

15. chloroquine.ti,ab.

16. quinine.ti,ab.

17. amodiaquine.ti,ab.

18. artesunate-amodiaquine.ti,ab.

19. (artesunate adj amodiaquine).ti,ab.

20. lumefantrine.ti,ab.

21. benflumetol.ti,ab.

22. artemether-lumefantine.ti,ab.

23. artemether-benflumetol.ti,ab.

24. (artemether adj lumefantrine).ti,ab.

25. (artemether adj benflumetol).ti,ab.

26. coartem.ti,ab.

27. halofantrine.ti,ab.

28. mefloquine.ti,ab.

29. artesunate-mefloquine.ti,ab.

30. (artesunate adj mefloquine).ti,ab.

31. primaquine.ti,ab.

32. sulfadoxine.ti,ab.

33. pyrimethamine.ti,ab.

34. (sulfadoxine adj pyrimethamine).ti,ab.

35. (pyrimethamine adj sulfadoxine).ti,ab.

36. sulfadoxine-pyrimethamine.ti,ab.

37. pyrimethamine-sulfadoxine.ti,ab.

38. amodiaquine/

39. amodiaquine plus artesunate/

40. amodiaquine plus primaquine/

41. artemether plus benflumetol/

42. artesunate plus mefloquine/

43. azithromycin plus chloroquine/

44. benflumetol/

45. chloroquine/

46. chloroquine plus primaquine/

47. dihydroartemisinin plus piperaquine/

48. halofantrine/

49. mefloquine/

50. piperaquine/

51. primaquine/

52. pyrimethamine/

53. pyrimethamine plus sulfadoxine/

54. quinine/

55. quinine formate/

56. quinine sulfate/

57. or/12-56

58. electrocardiography/

59. electrocardiography monitoring/

60. Holter monitoring/

61. exp electrocardiogram/

62. heart disease/co, di, dt, pd, si, th, cn

63. cardiotoxicity/co, di, dt, pd, si, th, cn

64. ECG abnormality/co, di, dt, pd, si, th, cn

65. heart arrhythmia/co, di, dt, pd, si, th, cn

66. exp heart muscle conduction disturbance/

67. exp abnormal blood pressure/

68. (QT OR QTc).ti,ab.

69. (QT adj (interval$ OR prolongation)).ti,ab.

70. (QTc adj (interval$ OR prolongation)).ti,ab.

71. (electrocardiogra$ OR ECG).ti,ab.

72. (cardiac adj toxicity).ti,ab.

73. cardiotoxicity.ti,ab.

74. safety.ti,ab.

75. (blood adj pressure).ti,ab.

76. or/58-75

77. 11 AND 57 AND 76

**Global health** (searched 22^nd^ October 2015)

1. malaria/

2. cerebral malaria/

3. Plasmodium falciparum/

4. Plasmodium vivax/

5. malaria.ti,ab.

6. falciparum.ti,ab.

7. vivax.ti,ab.

8. plasmodium.ti,ab.

9. or/1-8

10. dihydroartemisinin-piperaquine.ti,ab.

11. (dihydroartemisinin adj piperaquine).ti,ab.

12. piperaquine.ti,ab.

13. chloroquine.ti,ab.

14. quinine.ti,ab.

15. amodiaquine.ti,ab.

16. artesunate-amodiaquine.ti,ab.

17. (artesunate adj amodiaquine).ti,ab.

18. lumefantrine.ti,ab.

19. benflumetol.ti,ab.

20. artemether-lumefantine.ti,ab.

21. artemether-benflumetol.ti,ab.

22. (artemether adj lumefantrine).ti,ab.

23. (artemether adj benflumetol).ti,ab.

24. coartem.ti,ab.

25. halofantrine.ti,ab.

26. mefloquine.ti,ab.

27. artesunate-mefloquine.ti,ab.

28. (artesunate adj mefloquine).ti,ab.

29. primaquine.ti,ab.

30. sulfadoxine.ti,ab.

31. pyrimethamine.ti,ab.

32. (sulfadoxine adj pyrimethamine).ti,ab.

33. (pyrimethamine adj sulfadoxine).ti,ab.

34. sulfadoxine-pyrimethamine.ti,ab.

35. pyrimethamine-sulfadoxine.ti,ab.

36. halofantrine/

37. lumefantrine/

38. mefloquine/

39. piperaquine/

40. amodiaquine/

41. chloroquine/

42. primaquine/

43. pyrimethamine/

44. quinine/

45. sulfadoxine/

46. or/10-45

47. electrocardiography/

48. electrocardiograms/

49. drug toxicity/

50. cardiac rhythm/

51. heart rate/

52. arrhythmia/

53. hypotension/

54. pulse rate/

55. (QT OR QTc).ti,ab.

56. (QT adj (interval$ OR prolongation)).ti,ab.

57. (QTc adj (interval$ OR prolongation)).ti,ab.

58. (cardiac adj toxicity).ti,ab.

59. cardiotoxicity.ti,ab.

60. safety.ti,ab.

61. (blood adj pressure).ti,ab.

62. or/47-61

59. 9 AND 46 AND 62
