# Supplementary material for: The arrhythmogenic cardiotoxicity of the quinoline and structurally related antimalarial drugs: a systematic review
Source: BMC Med. 2018 Nov 7;16:200. doi: 10.1186/s12916-018-1188-2 (PMC6220451; doi:10.1186/s12916-018-1188-2)
Supplement: Supplementary file 2 — Complete list of variables extracted from articles. (DOCX 15 kb) [file 12916_2018_1188_MOESM2_ESM.docx]

| **Additional file 2** Complete list of variables extracted from articles |
| --- |
| General details |
| Title, year of publication, journal |
| Trial registry number |
| Details of sponsor/funding |
| Date of start and end of data collection |
| Geographic location of study (country and in-country location)  Primary aim of study (drug efficacy, cardiovascular safety, pharmacokinetic, other) |
| Trial design details |
| Trial design (including if randomised and blinding attempted) |
| Presence or absence of power calculation |
| Total number of participants recruited to study |
| Total number or participants analysed |
| Total number or participants who had ECGs |
| Trial population details |
| Number of males and females |
| Presence or absence of children and pregnant women included in trial |
| Mean and SD of age, median age, interquartile range, age range |
| Inclusion and exclusion criteria specifically detailing co-medication and comorbidities |
| Malaria species participants infected with, whether single or mixed infection |
| Severity of malaria infection |
| Method of diagnosis of malaria |
| Medication details |
| Medication(s) administered during trial |
| Route of administration of medication(s) |
| Medication manufacturer(s) |
| Level of supervision of drug administration |
| Length of follow up of trial |
| Pharmacokinetic sample details |
| Number of samples per participant for PK analysis |
| Time points PK samples collected |
| Sample matrix and assay used for PK analysis |
| ECG methodology details |
| Time points ECGs recorded |
| ECGs taken before or after drug administration |
| Definition(s) of prolonged QTc |
| Brand and type of ECG machine |
| Paper speed |
| Manual or automatic QT interval measurement |
| Details of investigator who measured QT interval  Whether there was any further independent measurement and calculation of QTc |
| Leads QT interval measured from |
| Definition of end of T wave |
| Correction formula used |
| Details of food intake around drug dosing |
| Position of patient when ECG recorded |
| Electrocardiographic intervals |
| Mean ± SD QTcB at baseline |
| Number of participants with prolonged QT intervals at baseline |
| Mean ± SD QTcB at 4 hours |
| Mean ± SD QTcB at 24 hours |
| Mean ± SD QTcB at 7 days |
| Number of participants who developed a prolonged QT interval during trial |
| Mean ± SD maximal change in QTcB from baseline |
| Mean ± SD maximal QTcB recorded |
| % mean ± SD maximal change in QTcB from baseline |
| Time point at which mean maximal change observed |
| Individual patient maximum change observed (QTcB and QTcF) |
| Any cardiac adverse events or other cases of note |
| ECG, electrocardiogram; SD, standard deviation; PK, pharmacokinetic; QTc, heart rate corrected QT interval; QTcB, Bazett’s-corrected QT interval; QTcF, Fridericia’s-corrected QT interval |
