# Supplementary material for: The arrhythmogenic cardiotoxicity of the quinoline and structurally related antimalarial drugs: a systematic review
Source: BMC Med. 2018 Nov 7;16:200. doi: 10.1186/s12916-018-1188-2 (PMC6220451; doi:10.1186/s12916-018-1188-2)
Supplement: Supplementary file 3 — List of primary and secondary outcomes. (DOCX 14 kb) [file 12916_2018_1188_MOESM3_ESM.docx]

| **Additional file 3** List of primary and secondary outcomes |
| --- |
| Primary outcomes: clinical features |
| Number and description of clinical cardiovascular AEs (palpitations, syncope, sudden cardiac death) |
| Number and description of life-threatening arrhythmias recorded by ECG (VT, VF, TdP) |
| Primary outcomes: electrocardiographic intervals |
| Mean ± SD QTcB at baseline, 4 hours, 24 hours, 7 days |
| Proportion of patients who developed a prolonged QT interval during trial |
| Mean ± SD maximal change in QTcB from baseline |
| Mean ± SD maximal QTcB recorded during trial |
| Mean ± SD maximal % change in QTcB from baseline |
| Mean ± SD time at which the maximal QTcB was recorded |
| Mean ± SD individual patient maximal QTcB or QTcF recorded |
| Secondary outcomes: electrocardiographic methodology |
| Mean and median number of ECGs collected per patient per trial |
| Proportion of trials using 1-lead, 12-lead or ambulatory ECGs |
| Proportion of trials using manual or automatic, or manual and automatic method of reading ECGs |
| Proportion of trials using Bazett’s, Fridericia’s or other heart rate correction method |
| Proportion of trials recording ECGs at 25mm/s or 50 mm/s |
| Proportion of trials specifying the lead the QT interval was measured from |
| Proportion of trials detailing the food intake of their participants around drug administration |
| Proportion of trials using the ICH definition of prolongation |
| Proportion of trials specifying a definition of QT prolongation used |
| Proportion of trials collecting pharmacokinetic data |
| Median number of pharmacokinetic samples collected per patient per trial |
| Primary and secondary outcomes determined for each of the 9 drugs included in the review. AEs, adverse events; ECG, electrocardiogram; VT, ventricular tachycardia; VF, ventricular fibrillation; TdP, Torsade de Pointes; SD, standard deviation; QTcB, Bazett’s-corrected QT interval; QTcF: Fridericia’s-corrected QT interval; ICH, International Conference on Harmonisation of Technical Requirements for Registration of Pharmaceuticals for Human Use |
