# Supplementary material for: The arrhythmogenic cardiotoxicity of the quinoline and structurally related antimalarial drugs: a systematic review
Source: BMC Med. 2018 Nov 7;16:200. doi: 10.1186/s12916-018-1188-2 (PMC6220451; doi:10.1186/s12916-018-1188-2)
Supplement: Supplementary file 4 — Criteria used for assessment of risk of bias of individual studies. (DOCX 17 kb) [file 12916_2018_1188_MOESM4_ESM.docx]

| **Additional file 4** Criteria used for assessment of risk of bias of individual studies | |
| --- | --- |
| **Blinding of participants and personnel** *Was knowledge of the allocated interventions adequately prevented during the study?* | |
| Low risk | - Blinding of participants and key study personnel; unlikely the blinding could have been broken |
| Unclear risk | - Insufficient information provided |
| High risk | - No blinding or incomplete blinding, and the outcome or outcome measurement is likely to be influenced by lack of blinding - Blinding of key study participants and personnel attempted, but likely that the blinding could have been broken |
| **Blinding of outcome assessors** *Were study personnel who performed or interpreted ECGs blinded?* | |
| Low risk | - Blinding of study personnel who performed or interpreted ECGs; unlikely the blinding could have been broken |
| Unclear risk | - Insufficient information provided |
| High risk | - No blinding or incomplete blinding, and the outcome measurement is likely to be influenced by lack of blinding |
| **Incomplete outcome data** *Were incomplete outcome data, specifically cardiovascular outcomes, adequately addressed?* | |
| Low risk | - No missing outcome data - Reasons for missing outcome data unlikely to be related to true outcome - Missing outcome data balanced in numbers across intervention groups, with similar reasons for missing data across groups |
| Unclear risk | - Insufficient reporting of attrition or exclusions (e.g. number randomised not stated, no reasons for missing data provided) |
| High risk | - Reason for missing outcome data likely to be related to true outcome, with either imbalance in numbers or reasons for missing data across intervention groups - ‘As-treated’ analysis done with substantial departure of the intervention received from that assigned at randomisation |
| **Selective outcome reporting** *Are reports of the study free of suggestion of selective outcome reporting?* | |
| Low risk | - The study protocol is available and the study’s pre-specified outcomes, with regards to cardiovascular safety, have been reported in the pre-specified way - The study protocol is not available but the published reports include all expected outcomes, including those that were pre-specified |
| Unclear risk | - Insufficient information provided |
| High risk | - Incomplete reporting of the study’s pre-specified outcomes with regards to cardiovascular safety - One or more reported outcomes, with regards to cardiovascular safety, were not pre-specified |
| **Other potential threats to validity** *Was the study apparently free of other problems that could put it at a risk of bias?* | |
| Low risk | - Instrument sensitivity: a 12-lead electrocardiogram was used to record cardiovascular parameters, recorded at 50mm/s - At least 2 ECGs were recorded within 12 hours from initial drug administration - Manually interpreted ECGs |
| Unclear risk | - Insufficient information |
| High risk | - 1 or 3 lead ECG used to record cardiovascular parameters, recorded at 25mm/s - < 2 ECGs were recorded within 12 hours from initial drug administration - Automatically interpreted ECGs |
| ECGs, electrocardiograms | |
