# Supplementary material for: The arrhythmogenic cardiotoxicity of the quinoline and structurally related antimalarial drugs: a systematic review
Source: BMC Med. 2018 Nov 7;16:200. doi: 10.1186/s12916-018-1188-2 (PMC6220451; doi:10.1186/s12916-018-1188-2)
Supplement: Supplementary file 5 — List of references included in the review. (DOCX 28 kb) [file 12916_2018_1188_MOESM5_ESM.docx]

**Additional file 5** List of references included in the review

1. Abdulla S, Amuri B, Kabanywanyi AM, Ubben D, Reynolds C, Pascoe S, et al. Early clinical development of artemether-lumefantrine dispersible tablet: palatability of three flavours and bioavailability in healthy subjects. Malar J. 2010;9:253.

2. Abdulla S, Sagara I, Borrmann S, D’Alessandro U, Gonzalez R, Hamel M, et al. Efficacy and safety of artemether-lumefantrine dispersible tablets compared with crushed commercial tablets in African infants and children with uncomplicated malaria: a randomised, single-blind, multicentre trial. Lancet. 2008;372:1819–27.

3. Adjei GO, Oduro-Boatey C, Rodrigues OP, Hoegberg LC, Alifrangis M, Kurtzhals JA, et al. Electrocardiographic study in Ghanaian children with uncomplicated malaria, treated with artesunate-amodiaquine or artemether-lumefantrine. Malar J. 2012;11:420.

4. Alecrim MG, Lacerda M V, Mourao MP, Alecrim WD, Padilha A, Cardoso BS, et al. Successful treatment of Plasmodium falciparum malaria with a six-dose regimen of artemether-lumefantrine versus quinine-doxycycline in the western Amazon region of Brazil. Am J Trop Med Hyg. 2006;74:20–5.

5. Ashley EA, Krudsood S, Phaiphun L, Srivilairit S, McGready R, Leowattana W, et al. Randomized, controlled dose-optimization studies of dihydroartemisinin-piperaquine for the treatment of uncomplicated multidrug-resistant falciparum malaria in Thailand. J Infect Dis. 2004;190:1773–82.

6. Assimadi JK, Gbadoe AD, Agbodjan-Djossou O, Ayewada K, Goeh-Akue E, Kusiaku K, et al. Treatment of cerebral malaria in African children by intravenous quinine: comparison of a loading dose regimen to a regimen without a loading dose. Arch Pédiatrie. 2002;9:587–94.

7. Auprayoon P, Sukontason K, Na-Bangchang K, Banmairuroi V, Molunto P, Karbwang J. Pharmacokinetics of quinine in chronic liver disease. Br J Clin Pharmacol. 1995;40:494–7.

8. Babalola CP, Kolade YT, Olaniyi AA, Adedapo A, Scriba GKE. Effect of fluconazole on the pharmacokinetics of halofantrine in healthy volunteers. J Clin Pharm Ther. 2009;34:677–82.

9. Baiden R, Oduro A, Halidou T, Gyapong M, Sie A, Macete E, et al. Prospective observational study to evaluate the clinical safety of the fixed-dose artemisinin-based combination Eurartesim (dihydroartemisinin/piperaquine), in public health facilities in Burkina Faso, Mozambique, Ghana, and Tanzania. Malar J. 2015;14.

10. Bassat Q, Mulenga M, Tinto H, Piola P, Borrmann S, Menendez C, et al. Dihydroartemisinin-piperaquine and artemether-lumefantrine for treating uncomplicated malaria in African children: a randomised, non-inferiority trial. PLoS One. 2009;4.

11. Bassi PU, Onyeji CO, Ukponmwan OE. Effects of tetracycline on the pharmacokinetics of halofantrine in healthy volunteers. Br J Clin Pharmacol. 2004;58:52–5.

12. Benjamin JM, Moore BR, Salman S, Page-Sharp M, Tawat S, Yadi G, et al. Population pharmacokinetics, tolerability, and safety of dihydroartemisinin-piperaquine and sulfadoxine-pyrimethamine-piperaquine in pregnant and nonpregnant Papua New Guinean women. Antimicrob Agents Chemother. 2015;59:4260–71.

13. Bethell DB, Phuong PT, Phuong CX, Nosten F, Waller D, Davis TM, et al. Electrocardiographic monitoring in severe falciparum malaria. Trans R Soc Trop Med Hyg. 1996;90:266–9.

14. Bhatt KM, Samia BM, Bhatt SM, Wasunna KM. Efficacy and safety of an artesunate/mefloquine combination (artequin) in the treatment of uncomplicated P. falciparum malaria in Kenya. East Afr Med J. 2006;83:236–42.

15. Bigira V, Kapisi J, Clark TD, Kinara S, Mwangwa F, Muhindo MK, et al. Protective efficacy and safety of three antimalarial regimens for the prevention of malaria in young Ugandan children: A randomized controlled trial. PLoS Med. 2014;11:e1001689.

16. Bindschedler M, Lefèvre G, Ezzet F, Schaeffer N, Meyer I, Thomsen MS. Cardiac effects of co-artemether (artemether/lumefantrine) and mefloquine given alone or in combination to healthy volunteers. Eur J Clin Pharmacol. 2000;56:375–81.

17. Bindschedler M, Lefèvre G, Degen P, Sioufi A. Comparison of the cardiac effects of the antimalarials co-artemether and halofantrine in healthy participants. Am J Trop Med Hyg. 2002;66:293–8.

18. Bouchaud O, Monlun E, Muanza K, Fontanet A, Scott T, Goetschel A, et al. Atovaquone plus proguanil versus halofantrine for the treatment of imported acute uncomplicated Plasmodium falciparum malaria in non-immune adults: A randomized comparative trial. Am J Trop Med Hyg. 2000;63:274–9.

19. Bouyou-Akotet MK, Ramharter M, Ngoungou EB, Mamfoumbi MM, Mihindou MP, Missinou MA, et al. Efficacy and safety of a new pediatric artesunate-mefloquine drug formulation for the treatment of uncomplicated falciparum malaria in Gabon. Wien Klin Wochenschr. 2010;122:173–8.

20. Bregani ER, Tien T Van, Cabibbe M, Figini G, Manenti F. Holter monitoring in children with severe Plasmodium falciparum malaria during IV quinine treatment. J Trop Pediatr. 2004;50:61.

21. Bunnag D, Harinasuta T, Looareesuwan S, Chittamas S, Pannavut W, Berthe J, et al. A combination of quinine, quinidine and cinchonine (LA 40221) in the treatment of chloroquine resistant falciparum malaria in Thailand: Two double-blind trials. Trans R Soc Trop Med Hyg. 1989;83:66.

22. Bunnag D, Karbwang J, Viravan C, Chitamas S, Harinasuta T. Clinical trials of mefloquine with tetracycline. Southeast Asian J Trop Med Public Health. 1992;23:377–82.

23. Byakika-Kibwika P, Lamorde M, Lwabi P, Nyakoojo WB, Okaba-Kayom V, Mayanja-Kizza H, et al. Cardiac Conduction Safety during Coadministration of Artemether-Lumefantrine and Lopinavir/Ritonavir in HIV-Infected Ugandan Adults. Chemother Res Pract. 2011;

24. Byakika-Kibwika P, Lamorde M, Okaba-Kayom V, Mayanja-Kizza H, Katabira E, Hanpithakpong W, et al. Lopinavir/ritonavir significantly influences pharmacokinetic exposure of artemether/lumefantrine in HIV-infected Ugandan adults. Chemother Res Pract. 2011;67:1217–23.

25. Chanthap L, Tsuyuoka R, Na-Bangchang K, Nivanna N, Suksom D, Sovannarith T, et al. Investigation of bioavailability, pharmacokinetics and safety of new pediatric formulations of artesunate and mefloquine. Southeast Asian J Trop Med Public Health. 2005;36:34–43.

26. Claessen FA, van Boxtel CJ, Perenboom RM, Tange RA, Wetsteijn JC, Kager PA. Quinine pharmacokinetics: Ototoxic and cardiotoxic effects in healthy Caucasian subjects and in patients with falciparum malaria. Trop Med Int Heal. 1998;3:482–9.

27. Cook JA, Randinitis EJ, Bramson CR, Wesche DL. Lack of a pharmacokinetic interaction between azithromycin and chloroquine. Am J Trop Med Hyg. 2006;74:407–12.

28. Darpo B, Ferber G, Siegl P, Laurijssens B, Macintyre F, Toovey S, et al. Evaluation of the QT effect of a combination of piperaquine and a novel anti-malarial drug candidate OZ439, for the treatment of uncomplicated malaria. Br J Clin Pharmacol. 2015;80:706–15.

29. Davis TM, Supanaranond W, Pukrittayakamee S, Karbwang J, Molunto P, Mekthon S, et al. A safe and effective consecutive-infusion regimen for rapid quinine loading in severe falciparum malaria. J Infect Dis. 1990;161:1305–8.

30. Davis TM, White NJ, Looareesuwan S, Silamut K, Warrell DA. Quinine pharmacokinetics in cerebral malaria: predicted plasma concentrations after a rapid intravenous loading using two-compartment model. Trans R Soc Trop Med Hyg. 1988;82:542–7.

31. de Souza JM. A phase I clinical trial of mefloquine in Brazilian male subjects. Bull World Health Organ. 1983;61:815–20.

32. de Souza JM. A phase II clinical trial of mefloquine in Brazilian male subjects. Bull World Health Organ. 1983;61:815–20.

33. de Souza JM, Sheth U., Wernsdorfer WH, Trigg PI, Doberstyn EB. A phase II/III double-blind, dose-finding clinical trial of a combination of mefloquine, sulfadoxine, and pyrimethamine (Fansimef) in falciparum malaria. Bull World Health Organ. 1987;65:357–61.

34. De Souza JM, Sheth UK, De Oliveira RMG, Roulet H, De Souza SD. An open, randomized, phase III clinical trial of mefloquine and of quinine plus sulfadoxine-pyrimethamine in the treatment of symptomatic falciparum malaria in Brazil. Bull World Health Organ. 1985;63:603–9.

35. de Souza JM, Sheth UK, de Oliveira RM, Gomes AT, Cavalcante EQ. A phase I clinical trial of Fansimef (mefloquine plus sulfadoxine-pyrimethamine) in Brazilian male subjects. Bull World Health Organ. 1985;63:611–5.

36. Edwards G, Looareesuwan S, Davies AJ, Wattanagoon Y, Phillips RE, Warrell DA. Pharmacokinetics of chloroquine in Thais: plasma and red-cell concentrations following an intravenous infusion to healthy subjects and patients with Plasmodium vivax malaria. Br J Clin Pharmacol. 1988;25:477–85.

37. Ekue JMK, Phiri DED, Sheth UK, Mukunyandela M. A double-blind trial of a fixed combination of mefloquine plus sulfadoxine-pyrimethamine compared with sulfadoxine-pyrimethamine alone in symptomatic falciparum malaria. Bull World Health Organ. 1987;65:369–73.

38. Ezzet F, van Vugt M, Nosten F, Looareesuwan S, White NJ. Pharmacokinetics and pharmacodynamics of lumefantrine (benflumetol) in acute falciparum malaria. Antimicrob Agents Chemother. 2000;44:697–704.

39. Falade CO, Ogunkunle OO, Dada-Adegbola HO, Falade AG, de Palacios PI, Hunt P, et al. Evaluation of the efficacy and safety of artemether-lumefantrine in the treatment of acute uncomplicated Plasmodium falciparum malaria in Nigerian infants and children. Malar J. 2008;7:246.

40. Falade C, Makanga M, Premji Z, Ortmann C-EE, Stockmeyer M, de Palacios PI. Efficacy and safety of artemether-lumefantrine (Coartem) tablets (six-dose regimen) in African infants and children with acute, uncomplicated falciparum malaria. Trans R Soc Trop Med Hyg. 2005;99:459–67.

41. Gargano N, Ubben D, Tommasini S, Bacchieri A, Corsi M, Bhattacharyya PC, et al. Therapeutic efficacy and safety of dihydroartemisinin-piperaquine versus artesunate-mefloquine in uncomplicated Plasmodium falciparum malaria in India. Malar J. 2012;11:233.

42. Gogtay NJ, Kamtekar KD, Dalvi SS, Mehta SS, Chogle AR, Aigal U, et al. A randomized, parallel study of the safety and efficacy of 45 mg primaquine versus 75 mg bulaquine as gametocytocidal agents in adults with blood schizonticide-responsive uncomplicated falciparum malaria. BMC Infect Dis. 2006;6:16.

43. Gustafsson L, Walker O, Alvan G, Beermann B, Estevez F, Gleisner L, et al. Disposition of chloroquine in man after single intravenous and oral doses. Br J Clin Pharmacol. 1983;15:471–9.

44. Hanboonkunupakarn B, Ashley EA, Jittamala P, Tarning J, Pukrittayakamee S, Hanpithakpong W, et al. Open-label crossover study of primaquine and dihydroartemisinin-piperaquine pharmacokinetics in healthy adult thai subjects. Antimicrob Agents Chemother. 2014;58:7340–6.

45. Harinasuta T, Bunnag D, Vanijanond S, Charoenlarp P, Suntharasmai P, Chitamas S, et al. Mefloquine, sulfadoxine, and pyrimethamine in the treatment of symptomatic falciparum malaria: a double-blind trial for determining the most effective dose. Bull World Health Organ. 1987;65:363–7.

46. Haroon N, Amichandwala K, Solu MG. Comparative efficacy of quinine and artesunate in the treatment of severe malaria: A randomized controlled trial. JK Sci. 2005;7:32–5.

47. Hatz C, Soto J, Nothdurft HD, Zoller T, Weitzel T, Loutan L, et al. Treatment of acute uncomplicated falciparum malaria with artemether-lumefantrine in non-immune populations: A safety, efficacy, and pharmacokinetic study. Am J Trop Med Hyg. 2008;78:241–7.

48. Hien TT, Day NPJ, Phu NH, Mai NTH, Chau TTN, Loc PP, et al. A controlled trial of artemether or quinine in Vietnamese adults with severe falciparum malaria. N Engl J Med. 1996;335:76–83.

49. Hien TT, Hanpithakpong W, Truong NT, Dung NT, Toi P Van, Farrar J, et al. Orally formulated artemisinin in healthy fasting vietnamese male subjects: A randomized, four-sequence, open-label, pharmacokinetic crossover study. Clin Ther. 2011;33:644–54.

50. Hombhanje FW, Kereu RK, Bulungol P, Paika R. Halofantrine in the treatment of uncomplicated falciparum malaria with a three-dose regimen in Papua New Guinea: a preliminary report. P N G Med J. 1998;41:23–9.

51. Huang L, Parikh S, Rosenthal PJ, Lizak P, Marzan F, Dorsey G, et al. Concomitant efavirenz reduces pharmacokinetic exposure to the antimalarial drug artemether-lumefantrine in healthy volunteers. J Acquir Immune Defic Syndr. 2012;61:310–6.

52. Humayun M, Haider I, Badshah A. The effect of quinine on QT interval in patients in a tertiary care hospital. J Postgrad Med Inst. 2013;27:20–5.

53. Jaspers CA, Hopperus Buma AP, van Thiel PP, van Hulst RA, Kager PA. Tolerance of mefloquine chemoprophylaxis in Dutch military personnel. Am J Trop Med Hyg. 1996;55:230–4.

54. Jittamala P, Pukrittayakamee S, Ashley E, Nosten F, Hanboonkunupakarn B, Lee S, et al. Pharmacokinetic interactions between primaquine and pyronaridine-artesunate in healthy adult Thai subjects. Antimicrob Agents Chemother. 2015;59:505–13.

55. Kakuda TN, DeMasi R, van Delft Y, Mohammed P. Pharmacokinetic interaction between etravirine or darunavir/ritonavir and artemether/lumefantrine in healthy volunteers: a two-panel, two-way, two-period, randomized trial. HIV Med. uda@its.jnj.com; 2013;14:421–9.

56. Karbwang J, Bangchang KN, Bunnag D, Harinasuta T. Pharmacokinetics and pharmacodynamics of mefloquine in Thai patients with acute falciparum malaria. Bull World Health Organ. 1991;69:207–12.

57. Karbwang J, Davis TME, Looareesuwan S, Molunto P, Bunnag D, White NJ. A comparison of the pharmacokinetic and pharmacodynamic properties of quinine and quinidine in healthy Thai males. Br J Clin Pharmacol. 1993;35:265–71.

58. Karbwang J, Laothavorn P, Sukontason K, Thiha T, Rimchala W, Na-Bangchang K, et al. Effect of artemether on electrocardiogram in severe falciparum malaria. Southeast Asian J Trop Med Public Health. 1997;28:472–5.

59. Karbwang J, Na Bangchang K, Back DJ, Bunnag D, Rooney W. Effect of tetracycline on mefloquine pharmacokinetics in Thai males. Eur J Clin Pharmacol. 1992;43:567–9.

60. Karbwang J, Na Bangchang K, Bunnag D, Harinasuta T, Laothavorn P. Cardiac effect of halofantrine. Lancet. 1993;342:501.

61. Karbwang J, Na Bangchang K, Thanavibul A, Back DJ, Bunnag D. Pharmacokinetics of mefloquine in the presence of primaquine. Eur J Clin Pharmacol. 1992;42:559–60.

62. Karbwang J, Na-Bangchang K, Thanavibul A, Ditta-in M, Harinasuta T. A comparative clinical trial of two different regimens of artemether plus mefloquine in multidrug resistant falciparum malaria. Trans R Soc Trop Med Hyg. 1995;89:296–8.

63. Karbwang J, Na-Bangchang K, Thanavibul A, Laothavorn P, Ditta-in M, Harinasuta T. A comparative clinical trial of artemether and the sequential regimen of artemether-mefloquine in multidrug resistant falciparum malaria. J Antimicrob Chemother. 1995;36:1079–83.

64. Karbwang J, Sukontason K, Rimchala W, Namsiripongpun W, Tin T, Auprayoon P, et al. Preliminary report: a comparative clinical trial of artemether and quinine in severe falciparum malaria. Southeast Asian J Trop Med Public Health. 1992;23:768–72.

65. Karbwang J, Thanavibul A, Molunto P, Na Bangchang K. The pharmacokinetics of quinine in patients with hepatitis. Br J Clin Pharmacol. 1993;35:444–6.

66. Karbwang J, Tin T, Rimchala W, Sukontason K, Namsiripongpun V, Thanavibul A, et al. Comparison of artemether and quinine in the treatment of severe falciparum malaria in south-east Thailand. Trans R Soc Trop Med Hyg. 1995;89:668–71.

67. Karunajeewa H, Lim C, Hung TY, Ilett KF, Denis MB, Socheat D, et al. Safety evaluation of fixed combination piperaquine plus dihydroartemisinin (Artekin) in Cambodian children and adults with malaria. Br J Clin Pharmacol. 2003;57:93–9.

68. Kayentao K, Doumbo OK, Pénali LK, Offianan AT, Bhatt KM, Kimani J, et al. Pyronaridine-artesunate granules versus artemether-lumefantrine crushed tablets in children with Plasmodium falciparum malaria: a randomized controlled trial. Malar J. 2012;11:364.

69. Khan MZ, Isani Z, Ahmed TM, Zafar AB, Gilal N, Maqbool S, et al. Efficacy and safety of halofantrine in Pakistani children and adults with malaria caused by P. falciparum and P. vivax. Southeast Asian J Trop Med Public Health. 2006;37:613–8.

70. Khan SJ, Munib S. Efficacy of Halofantrine Hydrochloride in vivax malaria. J Postgrad Med Inst. 2005;19:276–80.

71. Khan SJ, Shah N, Ali M. Efficacy of loading versus standard doses of quinine in cerebral malaria. Rawal Med J. 2011;36:86–8.

72. Kinde-Gazard D, Ogouyemi-Hounto A, Capo-Chichi L, Gbaguidi J, Massougbodji A. A randomized clinical trial comparing the effectiveness and tolerability of artemisinine-naphthoquine (Arco) and artemether-lumefantrine (Coartem) in the treatment of uncomplicated malaria in Benin. Bull la Société Pathol Exot. 2012;105:208–14.

73. Kredo T, Mauff K, Van der Walt JS, Wiesner L, Maartens G, Cohen K, et al. Interaction between artemether-lumefantrine and nevirapine-based antiretroviral therapy in HIV-1-infected patients. Antimicrob Agents Chemother. 2011;55:5616–23.

74. Krishna S, ter Kuile F, Supanaranond W, Pukrittayakamee S, Teja-Isavadharm P, Kyle D, et al. Pharmacokinetics, efficacy and toxicity of parenteral halofantrine in uncomplicated malaria. Br J Clin Pharmacol. 1993;36:585–91.

75. Krudsood S, Looareesuwan S, Tangpukdee N, Wilairatana P, Phumratanaprapin W, Leowattana W, et al. New fixed-dose artesunate-mefloquine formulation against multidrug-resistant Plasmodium falciparum in adults: a comparative phase IIb safety and pharmacokinetic study with standard-dose nonfixed artesunate plus mefloquine. Antimicrob Agents Chemother. 2010;54:3730–7.

76. Krudsood S, Looareesuwan S, Wilairatama P, Leowattana W, Tangpukdee N, Chalermrut K, et al. Effect of artesunate and mefloquine in combination on the Fridericia corrected QT intervals in Plasmodium falciparum infected adults from Thailand. Trop Med Int Heal. 2011;16:458–65.

77. Kshirsagar NA, Gogtay NJ, Moorthy NS, Garg MR, Dalvi SS, Chogle AR, et al. A randomized, double-blind, parallel-group, comparative safety, and efficacy trial of oral co-artemether versus oral chloroquine in the treatment of acute uncomplicated Plasmodium falciparum malaria in adults in India. Am J Trop Med Hyg. 2000;62:402–8.

78. Laman M, Moore BR, Benjamin JM, Yadi G, Bona C, Warrel J, et al. Artemisinin-Naphthoquine versus Artemether-Lumefantrine for Uncomplicated Malaria in Papua New Guinean Children: An Open-Label Randomized Trial. PLoS Med. 2014;11:e1001773.

79. Latha K, Ruckmani A. The effect of verapamil in malaria - a prospective randomized double blind control clinical study. J Clin Diagnostic Res. 2010;4:2707–13.

80. Lavallée I, Marc E, Moulin F, Treluyer JM, Imbert P, Gendrel D. Cardiac rhythm disturbances and prolongation of the QTc interval with halofantrine. Arch Pédiatrie. 2001;8:795–800.

81. Lefèvre G, Carpenter P, Souppart C, Schmidli H, Martin JM, Lane A, et al. Interaction trial between artemether-lumefantrine (Riamet) and quinine in healthy subjects. J Clin Pharmacol. 2002;42:1147–58.

82. Lefèvre G, Looareesuwan S, Treeprasertsuk S, Krudsood S, Silachamroon U, Gathmann I, et al. A clinical and pharmacokinetic trial of six doses of artemether-lumefantrine for multidrug-resistant Plasmodium falciparum malaria in Thailand. Am J Trop Med Hyg. 2001;64:247–56.

83. Lefèvre G, Bhad P, Jain JP, Kalluri S, Cheng Y, Dave H, et al. Evaluation of two novel tablet formulations of artemether-lumefantrine (Coartem) for bioequivalence in a randomized, open-label, two-period study. Malar J. 2013;12:312.

84. Lefèvre G, Carpenter P, Souppart C, Schmidli H, McClean M, Stypinski D. Pharmacokinetics and electrocardiographic pharmacodynamics of artemether-lumefantrine (Riamet) with concomitant administration of ketoconazole in healthy subjects. Br J Clin Pharmacol. 2002;54:485–92.

85. Liu Y, Hu C, Liu G, Jia J, Yu C, Zhu J, et al. A replicate designed bioequivalence study to compare two fixed-dose combination products of artesunate and amodiaquine in healthy Chinese volunteers. Antimicrob Agents Chemother. 2014;58:6009–15.

86. Llanos-Cuentas A, Lacerda M V, Rueangweerayut R, Krudsood S, Gupta SK, Kochar SK, et al. Tafenoquine plus chloroquine for the treatment and relapse prevention of Plasmodium vivax malaria (DETECTIVE): a multicentre, double-blind, randomised, phase 2b dose-selection study. Lancet. 2014;383:1049–58.

87. Lon C, Manning JE, Vanachayangkul P, So M, Sea D, Se Y, et al. Efficacy of two versus three-day regimens of dihydroartemisinin-piperaquine for uncomplicated malaria in military personnel in northern Cambodia: an open-label randomized trial. PLoS One. 2014;9.

88. Looareesuwan S, White NJ, Chanthavanich P, Edwards G, Nicholl D, Bunch C, et al. Cardiovascular toxicity and distribution kinetics of intravenous chloroquine. Br J Clin Pharmacol. 1986;22:31–6.

89. Manning J, Vanachayangkul P, Lon C, Spring M, So M, Sea D, et al. Randomized, double-blind, placebo-controlled clinical trial of a two-day regimen of dihydroartemisinin-piperaquine for malaria prevention halted for concern over prolonged corrected QT interval. Antimicrob Agents Chemother. 2014;58:6056–67.

90. Mansor SM, Taylor TE, McGrath CS, Edwards G, Ward SA, Wirima JJ, et al. The safety and kinetics of intramuscular quinine in Malawian children with moderately severe falciparum malaria. Trans R Soc Trop Med Hyg. 1990;84:482–7.

91. Massougbodji A, Kone M, Kinde-Gazard D, Same-Ekobo A, Cambon N, Mueller EA. A randomized, double-blind study on the efficacy and safety of practical three-day regimen with artesunate and mefloquine for the treatment of uncomplicated Plasmodium falciparum malaria in Africa. Trans R Soc Trop Med Hyg. 2002;96:655–9.

92. Matson PA, Luby SP, Redd SC, Rolka HR, Meriwether RA. Cardiac effects of standard-dose halofantrine therapy. Am J Trop Med Hyg. 1996;54:229–31.

93. Mayxay M, Keomany S, Khanthavong M, Souvannasing P, Stepniewska K, Khomthilath T, et al. A phase III, randomized, non-inferiority trial to assess the efficacy and safety of dihydroartemisinin-piperaquine in comparison with artesunate- mefloquine in patients with uncomplicated Plasmodium falciparum Malaria in Southern Laos. Am J Trop Med Hyg. 2010;83:1221–9.

94. McGready R, Stepniewska K, Lindegardh N, Ashley EA, La Y, Singhasivanon P, et al. The pharmacokinetics of artemether and lumefantrine in pregnant women with uncomplicated falciparum malaria. Eur J Clin Pharmacol. 2006;62:1021–31.

95. McGready R, Tan SO, Ashley EA, Pimanpanarak M, Viladpai-Nguen J, Phaiphun L, et al. A randomised controlled trial of artemether-lumefantrine versus artesunate for uncomplicated plasmodium falciparum treatment in pregnancy. PLoS Med. 2008;5:1699–715.

96. Miller AK, Harrell E, Ye L, Baptiste-Brown S, Kleim J-P, Ohrt C, et al. Pharmacokinetic interactions and safety evaluations of coadministered tafenoquine and chloroquine in healthy subjects. Br J Clin Pharmacol. 2013;76:858–67.

97. Miller RS, Wongsrichanalai C, Buathong N, McDaniel P, Walsh DS, Knirsch C, et al. Effective treatment of uncomplicated Plasmodium falciparum malaria with azithromycin-quinine combinations: a randomized, dose-ranging study. Am J Trop Med Hyg. 2006;74:401–6.

98. Minodier P, Noel G, Salles M, Retornaz K, Walters H, Combes JC, et al. Mefloquine versus halofantrine in children suffering from acute uncomplicated falciparum malaria. Arch Pédiatrie. 2005;12:67–71.

99. Monlun E, Le Metayer P, Szwandt S, Neau D, Longy-Boursier M, Horton J, et al. Cardiac complications of halofantrine: a prospective study of 20 patients. Trans R Soc Trop Med Hyg. 1995;89:430–3.

100. Moore BR, Benjamin JM, Salman S, Griffin S, Ginny E, Page-Sharp M, et al. Effect of coadministered fat on the tolerability, safety, and pharmacokinetic properties of dihydroartemisinin-piperaquine in Papua New Guinean children with uncomplicated malaria. Antimicrob Agents Chemother. 2014;58:5784–94.

101. Mra R, Myint PT, Shwe Ti. Electrocardiographic effects of quinine and quinidine in the treatment of falciparum malaria. Myanmar Heal Sci Res J. 1991;3:1–5.

102. Murphy S, English M, Waruiru C, Mwangi I, Amukoye E, Crawley J, et al. An open randomized trial of artemether versus quinine in the treatment of cerebral malaria in African children. Trans R Soc Trop Med Hyg. 1996;90:298–301.

103. Mutabingwa TK, Muze K, Ord R, Briceno M, Greenwood BM, Drakeley C, et al. Randomized trial of artesunate+amodiaquine, sulfadoxine-pyrimethamine+amodiaquine, chlorproguanal-dapsone and SP for malaria in pregnancy in Tanzania. PLoS One. 2009;4:e5138.

104. Myint PT, Shwe T. A controlled clinical trial of artemether (qinghaosu derivative) versus quinine in complicated and severe falciparum malaria. Trans R Soc Trop Med Hyg. 1987;81:559–61.

105. Mytton OT, Ashley EA, Peto L, Price RN, La Y, Hae R, et al. Short Report: Electrocardiographic safety evaluation of dihydroartemisinin piperaquine in the treatment of uncomplicated falciparum malaria. Am J Trop Med Hyg. 2007;77:447–50.

106. Mzayek F, Deng H, Mather FJ, Wasilevich EC, Liu H, Hadi CM, et al. Randomized dose-ranging controlled trial of AQ-13, a candidate antimalarial, and chloroquine in healthy volunteers. PLoS Clin Trials. 2007;2.

107. Na-Bangchang K, Karbwang J, Palacios PA, Ubalee R, Saengtertsilapachai S, Wernsdorfer WH. Pharmacokinetics and bioequivalence evaluation of three commercial tablet formulations of mefloquine when given in combination with dihydroartemisinin in patients with acute uncomplicated falciparum malaria. Eur J Cinical Pharmacol. 2000;55:743–8.

108. Na-Bangchang K, Limpaibul L, Thanavibul A, Tan-Ariya P, Karbwang J. The pharmacokinetics of chloroquine in healthy Thai subjects and patients with Plasmodium vivax malaria. Br J Clin Pharmacol. 1994;38:278–81.

109. Na-Bangchang K, Thanavibul A, Tippawangkosol P, Karbwang J. Pharmacokinetics of the four combination regimens of dihydroartemisinin/mefloquine in acute uncomplicated falciparum malaria. Southeast Asian J Trop Med Public Health. 2005;36:23–33.

110. Nasveld PE, Edstein MD, Reid M, Brennan L, Harris IE, Kitchener SJ, et al. Randomized, double-blind study of the safety, tolerability, and efficacy of tafenoquine versus mefloquine for malaria prophylaxis in nonimmune subjects. Antimicrob Agents Chemother. 2010;54:792–8.

111. Navaratnam V, Ramanathan S, Wahab MSA, Siew Hua G, Mansor SM, Kiechel J-R, et al. Tolerability and pharmacokinetics of non-fixed and fixed combinations of artesunate and amodiaquine in Malaysian healthy normal volunteers. Eur J Clin Pharmacol. 2009;65:809–21.

112. Ndiaye J-LA, Faye B, Gueye A, Tine R, Ndiaye D, Tchania C, et al. Repeated treatment of recurrent uncomplicated Plasmodium falciparum malaria in Senegal with fixed-dose artesunate plus amodiaquine versus fixed-dose artemether plus lumefantrine: A randomized, open-label trial. Malar J. 2011;10:237.

113. Newton PN, Chierakul W, Ruangveerayuth R, Silamut K, Teerapong P, Krudsood S, et al. A comparison of artesunate alone with combined artesunate and quinine in the parenteral treatment of acute falciparum malaria. Trans R Soc Trop Med Hyg. 2001;95:519–23.

114. Ngouesse B, Basco LK, Ringwald P, Keundjian A, Blackett KN. Cardiac effects of amodiaquine and sulfadoxine-pyrimethamine in malaria-infected african patients. Am J Trop Med Hyg. 2001;65:711–6.

115. Noedl H, Krudsood S, Chalermratana K, Silachamroon U, Leowattana W, Tangpukdee N, et al. Azithromycin combination therapy with artesunate or quinine for the treatment of uncomplicated Plasmodium falciparum malaria in adults: A randomized, phase 2 clinical trial in Thailand. Clin Infect Dis. United States; 2006;43:1264–71.

116. Nosten F, Karbwang J, White NJ, Honeymoon, Na Bangchang K, Bunnag D, et al. Mefloquine antimalarial prophylaxis in pregnancy: dose finding and pharmacokinetic study. Br J Clin Pharmacol. 1990;30:79–85.

117. Nosten F, Luxemburger C, ter Kuile FO, Woodrow C, Eh JP, Chongsuphajaisiddhi T, et al. Treatment of multidrug-resistant Plasmodium falciparum malaria with 3-day artesunate-mefloquine combination. J Infect Dis. 1994;170:971–7.

118. Nosten F, ter Kuile FO, Luxemburger C, Woodrow C, Kyle DE, Chongsuphajaisiddhi T, et al. Cardiac effects of antimalarial treatment with halofantrine. Lancet. 1993;341:1054–6.

119. Nosten F, ter Kuile F, Maelankiri L, Chongsuphajaisiddhi T, Nopdonrattakoon L, Tangkitchot S, et al. Mefloquine prophylaxis prevents malaria during pregnancy: a double-blind, placebo-controlled study. J Infect Dis. 1994;169:595–603.

120. Nyunt MM, Lu Y, El-Gasim M, Parsons TL, Petty BG, Hendrix CW. Effects of ritonavir-boosted lopinavir on the pharmacokinetics of quinine. Clin Pharmacol Ther. United States; 2012;91:889–95.

121. Ogunkunlke OO, Fehintola FA, Ogungbamigbe TO, Falade CO. Comparative cardiac effects of chlorproguanil/dapsone and chloroquine during treatment of acute uncomplicated falciparum malaria infection in Nigerian children. African J Biomed Res. 2011;14:161–7.

122. Ogutu B, Juma E, Obonyo C, Jullien V, Carn G, Vaillant M, et al. Fixed dose artesunate amodiaquine - A phase IIb, randomized comparative trial with non-fixed artesunate amodiaquine. Malar J. 2014;13.

123. Omoruyi SI, Onyeji CO, Daniyan MO. Effects of prior administration of amodiaquine on the disposition of halofantrine in healthy volunteers. Ther Drug Monit. 2007;29:203–6.

124. Orrell C, Little F, Smith P, Folb P, Taylor W, Olliaro P, et al. Pharmacokinetics and tolerability of artesunate and amodiaquine alone and in combination in healthy volunteers. Eur J Clin Pharmacol. 2008;64:683–90.

125. Phuong CXT, Bethell DB, Phuong PT, Mai TTT, Thuy TTN, Ha NTT, et al. Comparison of artemisinin suppositories, intramuscular artesunate and intravenous quinine for the treatment of severe childhood malaria. Trans R Soc Trop Med Hyg. 1997;91:335–42.

126. Piola P, Nabasumba C, Turyakira E, Dhorda M, Lindegardh N, Nyehangane D, et al. Efficacy and safety of artemether-lumefantrine compared with quinine in pregnant women with uncomplicated Plasmodium falciparum malaria: an open-label, randomised, non-inferiority trial. Lancet Infect Dis. United States; 2010;10:762–9.

127. Poravuth Y, Socheat D, Rueangweerayut R, Uthaisin C, Pyae Phyo A, Valecha N, et al. Pyronaridine-artesunate versus chloroquine in patients with acute Plasmodium vivax Malaria: A randomized, double-blind, non-inferiority trial. PLoS One. 2011;6:e14501.

128. Price RN, Nosten F, Luxemburger C, Kham A, Brockman A, Chongsuphajaisiddhi T, et al. Artesunate versus artemether in combination with mefloquine for the treatment of multidrug-resistant falciparum malaria. Trans R Soc Trop Med Hyg. 1995;89:523–7.

129. Pukrittayakamee S, Tarning J, Jittamala P, Charunwatthana P, Lawpoolsri S, Lee SJ, et al. Pharmacokinetic interactions between primaquine and chloroquine. Antimicrob Agents Chemother. 2014;58:3354–9.

130. Pyar KP, Myint WW, Kyaw MP, Zin T, Nyo K, Htut T, et al. Comparison of efficacy and safety of different brands of oral artesunate plus mefloquine in uncomplicated falciparum malaria in adults. Myanmar Heal Sci Res J. 2007;19:63–8.

131. Pyar KP, Myint WW, Kyaw MP, Zin T, Than M. Efficacy and safety of artemisinin-piperaquine (Artequick) compared to dihydroartemisinin-piperaquine (Artekin) in uncomplicated falciparum malaria in adults. Myanmar Heal Sci Res J. 2009;21:78–82.

132. Rasheed A, Saeed S. In vivo efficacy and safety of quinine-doxycycline combination in acute Plasmodium falciparum malaria. Pakistan J Med Sci. 2008;24:684–8.

133. Restrepo M, Botero D, Marquez RE, Boudreau EF, Navaratnam V. A clinical trial with halofantrine on patients with falciparum malaria in Colombia. Bull World Health Organ. 1996;74:591–7.

134. Roggelin L, Pelletier D, Hill JN, Feldt T, Hoffmann S, Ansong D, et al. Disease-associated QT-shortage versus quinine associated QT-prolongation: Age dependent ECG-effects in Ghanaian children with severe malaria. Malar J. 2014;13:219.

135. Roshammar D, Hai TN, Friberg Hietala S, Van Huong N, Ashton M. Pharmacokinetics of piperaquine after repeated oral administration of the antimalarial combination CV8 in 12 healthy male subjects. Eur J Clin Pharmacol. 2006;62:335–41.

136. Rueangweerayut R, Phyo AP, Uthaisin C, Poravuth Y, Binh TQ, H. T, et al. Pyronaridine-artesunate versus mefloquine plus artesunate for malaria. N Engl J Med. 2012;366:1298–309.

137. Sabchareon A, Chongsuphajaisiddhi T, Sinhasivanon V, Chanthavanich P, Attanath P. In vivo and in vitro responses to quinine and quinidine of Plasmodium falciparum. Bull World Health Organ. 1988;66:347–52.

138. Shwe T, Myint PT, Htut Y, Myint W, Soe L. The effect of mefloquine-artemether compared with quinine on patients with complicated falciparum malaria. Trans R Soc Trop Med Hyg. 1988;82:665–6.

139. Siriez J-Y, Lupoglazoff J-M, Bouchy-Bagros M-L, Pull L, Denjoy I. Effect of halofantrine on QT interval in children. Pathog Glob Health. 2012;106:124–5.

140. Song J, Socheat D, Tan B, Seila S, Xu Y, Ou F, et al. Randomized trials of artemisinin-piperaquine, dihydroartemisinin- piperaquine phosphate and artemether-lumefantrine for the treatment of multi-drug resistant falciparum malaria in Cambodia-Thailand border area. Malar J. 2011;10.

141. Sowunmi A, Falade CO, Oduola AM, Ogundahunsi OA, Fehintola FA, Gbotosho GO, et al. Cardiac effects of halofantrine in children suffering from acute uncomplicated falciparum malaria. Trans R Soc Trop Med Hyg. 1998;92:446–8.

142. Sowunmi A, Fehintola FA, Ogundahunsi OA, Ofi AB, Happi TC, Oduola AM. Comparative cardiac effects of halofantrine and chloroquine plus chlorpheniramine in children with acute uncomplicated falciparum malaria. Trans R Soc Trop Med Hyg. 1999;93:78–83.

143. Sowunmi A, Salako LA, Laoye OJ, Aderounmu AF. Combination of quinine, quinidine and cinchonine for the treatment of acute falciparum malaria: Correlation with the susceptibility of Plasmodium falciparum to the cinchona alkaloids in vitro. Trans R Soc Trop Med Hyg. 1990;84:626–9.

144. Stein DS, Jain JP, Kangas M, Lefèvre G, Machineni S, Griffin P, et al. Open-label, single-dose, parallel-group study in healthy volunteers to determine the drug-drug interaction potential between KAE609 (cipargamin) and piperaquine. Antimicrob Agents Chemother. 2015;59:3493–500.

145. Sukontason K, Karbwang J, Rimchala W, Tin T, Na-Bangchang K, Banmairuroi V, et al. Plasma quinine concentrations in falciparum malaria with acute renal failure. Trop Med Int Heal. 1996;1:236–42.

146. Supanaranond W, Davis TM, Pukrittayakamee S, Nagachinta B, White NJ. Abnormal circulatory control in falciparum malaria: the effects of antimalarial drugs. Eur J Clin Pharmacol. 1993;44:325–9.

147. Supanaranond W, Suputtamongkol Y, Davis TM, Pukrittayakamee S, Teja-Isavadharm P, Webster HK, et al. Lack of a significant adverse cardiovascular effect of combined quinine and mefloquine therapy for uncomplicated malaria. Trans R Soc Trop Med Hyg. 1997;91:694–6.

148. Taylor TE, Wills BA, Courval JM, Molyneux ME. Intramuscular artemether vs intravenous quinine: an open, randomized trial in Malawian children with cerebral malaria. Trop Med Int Heal. 1998;3:3–8.

149. ter Kuile FO, Nosten F, Luxemburger C, Kyle D, Teja-Isavatharm P, Phaipun L, et al. Mefloquine treatment of acute falciparum malaria: a prospective study of non-serious adverse effects in 3673 patients. Bull World Health Organ. 1995;73:631–42.

150. Thapa S, Hollander J, Linehan M, Cox-Singh J, Bista MB, Thakur GD, et al. Comparison of artemether-lumefantrine with sulfadoxine-pyrimethamine for the treatment of uncomplicated falciparum malaria in eastern Nepal. Am J Trop Med Hyg. 2007;77:423–30.

151. Thuma PE, Bhat GJ, Mabeza GF, Osborne C, Biemba G, Shakankale GM, et al. A randomized controlled trial of artemotil (beta-arteether) in Zambian children with cerebral malaria. Am J Trop Med Hyg. 2000;62:524–9.

152. Thuy LTD, Hung LN, Hung NC, Na-Bangchang K. Pharmacokinetics of mefloquine with dihydroartemisinin as 2-day regimens in patients with uncomplicated falciparum malaria. Southeast Asian J Trop Med Public Health. 2007;38:205–12.

153. Tjitra E, Hasugian AR, Siswantoro H, Prasetyorini B, Ekowatiningsih R, Yusnita EA, et al. Efficacy and safety of artemisinin-naphthoquine versus dihydroartemisinin- piperaquine in adult patients with uncomplicated malaria: A multi-centre study in Indonesia. Malar J. 2012;11:153.

154. Toure OA, Valecha N, Tshefu AK, Thompson R, Krudsood S, Gaye O, et al. A Phase 3, Double-Blind, Randomized Study of Arterolane Maleate-Piperaquine Phosphate vs Artemether-Lumefantrine for Falciparum Malaria in Adolescent and Adult Patients in Asia and Africa. Clin Infect Dis. 2016;62:964–71.

155. Touze JE, Bernard J, Keundjian A, Imbert P, Viguier A, Chaudet H, et al. Electrocardiographic changes and halofantrine plasma level during acute falciparum malaria. Am J Trop Med Hyg. 1996;54:225–8.

156. Touze JE, Heno P, Fourcade L, Deharo JC, Thomas G, Bohan S, et al. The effects of antimalarial drugs on ventricular repolarization. Am J Trop Med Hyg. 2002;67:54–60.

157. Trung TN, Tan B, van Phuc D, Song JP. A randomized, controlled trial of artemisinin-piperaquine vs dihydroartemisinin-piperaquine phosphate in treatment of falciparum malaria. Chin J Integr Med. 2009;15:189–92.

158. Tshefu AK, Gaye O, Kayentao K, Thompson R, Bhatt KM, Sesay SSS, et al. Efficacy and safety of a fixed-dose oral combination of pyronaridine-artesunate compared with artemether-lumefantrine in children and adults with uncomplicated Plasmodium falciparum malaria: a randomised non-inferiority trial. Lancet. 2010;375:1457–67.

159. Valecha N, Phyo AP, Mayxay M, Newton PM, Krudsood S, Keomany S, et al. An open-label, randomised study of dihydroartemisinin-piperaquine versus artesunate-mefloquine for falciparum malaria in Asia. PLoS One. 2010;5.

160. Valecha N, Krudsood S, Tangpukdee N, Mohanty S, Sharma SK, Tyagi PK, et al. Arterolane maleate plus piperaquine phosphate for treatment of uncomplicated plasmodium falciparum malaria: A comparative, multicenter, randomized clinical trial. Clin Infect Dis. 2012;55:663–71.

161. van Agtmael M, Bouchaud O, Malvy D, Delmont J, Danis M, Barette S, et al. The comparative efficacy and tolerability of CGP 56697 (artemether + lumefantrine) versus halofantrine in the treatment of uncomplicated falciparum malaria in travellers returning from the tropics to the Netherlands and France. Int J Antimicrob Agents. 1999;12:159–69.

162. van Hensbroek MB, Kwiatkowski D, van den Berg B, Hoek FJ, van Boxtel CJ, Kager PA. Quinine pharmacokinetics in young children with severe malaria. Am J Trop Med Hyg. 1996;54:237–42.

163. van Vugt M, Ezzet F, Nosten F, Gathmann I, Wilairatana P, Looareesuwan S, et al. No evidence of cardiotoxicity during antimalarial treatment with artemether-lumefantrine. Am J Trop Med Hyg. 1999;61:964–7.

164. Van Vugt M, Looareesuwan S, Wilairatana P, McGready R, Villegas L, Gathmann I, et al. Artemether-lumefantrine for the treatment of multidrug-resistant falciparum malaria. Trans R Soc Trop Med Hyg. 2000;94:545–8.

165. van Vugt M, Wilairatana P, Gemperli B, Gathmann I, Phaipun L, Brockman A, et al. Efficacy of six doses of artemether-lumefantrine (benflumetol) in multidrug-resistant Plasmodium falciparum malaria. Am J Trop Med Hyg. 1999;60:936–42.

166. von Seidlein L, Jaffar S, Greenwood B. Prolongation of the QTc interval in African children treated for falciparum malaria. Am J Trop Med Hyg. 1997;56:494–7.

167. von Seidlein L, Jaffar S, Pinder M, Haywood M, Snounou G, Gemperli B, et al. Treatment of African children with uncomplicated falciparum malaria with a new antimalarial drug, CGP 56697. J Infect Dis. 1997;176:1113–6.

168. Walker O, Salako LA, Omokhodion SI, Sowunmi A. An open randomized comparative study of intramuscular artemether and intravenous quinine in cerebral malaria in children. Trans R Soc Trop Med Hyg. 1993;87:564–6.

169. Watt G, Na-Nakorn A, Bateman DN, Plubha N, Mothanaprakoon P, Edstein M, et al. Amplification of quinine cardiac effects by the resistance-reversing agent prochlorperazine in falciparum malaria. Am J Trop Med Hyg. 1993;49:645–9.

170. Wattanagoon Y, Phillips RE, Warrell DA, Silamut K, Looareesuwan S, Nagachinta B, et al. Intramuscular loading dose of quinine for falciparum malaria: pharmacokinetics and toxicity. Br Med J. ENGLAND; 1986;293:11–3.

171. White NJ, Chanthavanich P, Krishna S, Bunch C, Silamut K. Quinine disposition kinetics. Br J Clin Pharmacol. 1983;16:399–403.

172. White NJ, Looareesuwan S, Edwards G. Pharmacokinetics of intravenous amodiaquine. Br J Clin Pharmacol. 1987;23:127–35.

173. White NJ, Looareesuwan S, Warrell DA. Quinine pharmacokinetics and toxicity in cerebral and uncomplicated falciparum malaria. Am J Med. 1982;73:564–72.

174. White NJ, Looareesuwan S, Warrell DA. Quinine and quinidine: a comparison of EKG effects during the treatment of malaria. J Cardiovasc Pharmacol. 1983;5:173–5.

175. White NJ, Looareesuwan S, Warrell DA, Warrell MJ, Chanthavanich P, Bunnag D, et al. Quinine loading dose in cerebral malaria. Am J Trop Med Hyg. 1983;32:1–5.

176. White NJ, Miller KD, Churchill FC, Berry C, Brown J, Williams SB, et al. Chloroquine treatment of severe malaria in children. Pharmacokinetics, toxicity, and new dosage recommendations. N Engl J Med. 1988;319:1493–500.

177. Win K, Than M, Thwe Y. Comparison of combinations of parenteral artemisinin derivatives plus oral mefloquine with intravenous quinine plus oral tetracycline for treating cerebral malaria. Bull World Health Organ. 1992;70:777–82.
