# Supplementary material for: The arrhythmogenic cardiotoxicity of the quinoline and structurally related antimalarial drugs: a systematic review
Source: BMC Med. 2018 Nov 7;16:200. doi: 10.1186/s12916-018-1188-2 (PMC6220451; doi:10.1186/s12916-018-1188-2)
Supplement: Supplementary file 6 — Number of studies conducted in each region, for each drug. (DOCX 14 kb) [file 12916_2018_1188_MOESM6_ESM.docx]

| **Additional file 6** Number of studies conducted in each region, for each drug | | | | | | | | | |
| --- | --- | --- | --- | --- | --- | --- | --- | --- | --- |
|  | **Quinine (51 studies)** | **Mefloquine (45 studies)** | **Lumefantrine (39 studies)** | **Piperaquine (24 studies)** | **Halofantrine (22 studies)** | **Chloroquine (17 studies)** | **SP (14 studies)** | **Amodiaquine (10 studies)** | **Primaquine (7 studies)** |
| S. E. Asia | 28 | 32 | 12 | 16 | 3 | 7 | 3 | 1 | 5 |
| S. Asia, E. Asia | 4 | 3 | 5 | 4 | 2 | 4 | 1 | 1 | 1 |
| E. Africa, W. Africa, S. Africa, Middle Africa | 13 | 8 | 25 | 8 | 5 | 4 | 4 | 6 | 0 |
| E. Europe, N. Europe, W. Europe | 2 | 2 | 7 | 1 | 7 | 0 | 0 | 0 | 0 |
| Americas, S. America, N. America | 2 | 5 | 3 | 0 | 3 | 3 | 5 | 0 | 1 |
| Melanesia | 0 | 0 | 1 | 2 | 1 | 0 | 1 | 0 | 0 |
| Missing data | 4 | 0 | 1 | 1 | 2 | 3 | 0 | 2 | 0 |

Geographical regions defined by the United Nation Statistics Division. SP, sulfadoxine-pyrimethamine.
