# Supplementary material for: The arrhythmogenic cardiotoxicity of the quinoline and structurally related antimalarial drugs: a systematic review
Source: BMC Med. 2018 Nov 7;16:200. doi: 10.1186/s12916-018-1188-2 (PMC6220451; doi:10.1186/s12916-018-1188-2)
Supplement: Supplementary file 7 — Other cardiovascular events observed, organised by arrhythmia. (DOCX 17 kb) [file 12916_2018_1188_MOESM7_ESM.docx]

**Additional file 7** Other cardiovascular events observed, organised by arrhythmia

| **Drug, reference** | | **Detail of arrhythmia** |
| --- | --- | --- |
| **First degree heart block** | | |
| Quinine | Miller, 2006 | 1/50 developed transient 1 ^st^ degree heart block in quinine-azithromycin group; 0/10 in quinine-doxycycline comparator group |
| Quinine | Sabchareon, 1988 | 56 events of prolonged PR in 33 patients over 7 days, 16 significantly so; 67 events in 33 patients in quinidine comparator group |
| Mefloquine, SP | Bunnag, 1992 | 1/24 developed 1^st^ degree heart block in mefloquine group; 2/26 in mefloquine+sulfadoxine+pyrimethamine comparator group |
| Mefloquine, SP | Harinasuta, 1987 | 4/46 transient and symptomless 1 ^st^ degree heart block following moderate dose mefloquine+sulfadoxine-pyrimethamine; 0/48 in higher dose comparator group |
| Halofantrine | Sowunmi, 1998 | 9 events of the PR interval exceeding 180 ms over 48 hours in 42 patients; no comparator group |
| Amodiaquine | Liu, 2014 | 2 events of 1^st^ degree heart block in 36 patients but no further comment on clinical significance (1 event in each of the two amodiaquine formulation groups, cross-over study) |
| Halofantrine | Karbwang, 1993(c) | 1/29 developed 1 ^st^ degree heart block in the acute infection group; 0/6 in the healthy control group |
| Halofantrine, mefloquine | Nosten, 1993 | 15/51 developed 1^st^ degree heart block following halofantrine; 3/10 in those treated with mefloquine followed by halofantrine |
| **Second degree heart block** | | |
| Halofantrine | Sowunmi, 1998 | In one child (1/42), Mobitz type I recorded at 48 hours following the start of treatment; no comparator group |
| Halofantrine, mefloquine | Nosten, 1993 | A 13-year old boy developed symptomless fluctuating Mobitz 1 and 2 block which resolved after treatment with halofantrine ceased (1/61 treated with halofantrine; 0/53 treated with mefloquine) |
| **Bradycardia** | | |
| Mefloquine | Win, 1992 | Bradycardia between 52 – 60 beats per minute with spontaneous recovery observed in ‘some patients’ of 24 treated with IV artesunate+mefloquine group; 0/50 in IM artemether+mefloquine group and 0/67 in quinine group |
| Mefloquine | Ter Kuile, 1995 | Bradycardia developed in 11 of 43 adults and 1 of 31 children by day 7 of treatment in those who underwent ECG monitoring (treatment groups pooled for analysis, all treated with mefloquine) |
| Mefloquine, SP | Bunnag, 1992 | 15/24 patients developed sinus bradycardia following mefloquine; 12/26 in mefloquine+sulfadoxine+pyrimethamine comparator group |
| Mefloquine | Karbwang, 1991 | 3/10 developed sinus bradycardia in lower dose mefloquine group; 4/10 in higher dose mefloquine comparator group |
| Mefloquine, SP | Harinasuta, 1987 | 24/46 developed symptomless bradycardia following moderate dose mefloquine+sulfadoxine-pyrimethamine; 22/48 in higher dose comparator group |
| Lumefantrine | Tshefu, 2010 | 5/423 developed mild bradycardia after treatment with lumefantrine; 5/849 in pyronaridine-artesunate comparator group |
| Amodiaquine | Adjei, 2012 | 7/47 developed bradycardia following amodiaquine; 0/30 in lumefantrine comparator group |
| Amodiaquine, SP | Ngouesse, 2001 | 14/20 and 16/20 developed bradycardia following amodiaquine on days 2 and 3 respectively, and in 12 patients on day 7; 2/8 in SP group on day 3 |
| Halofantrine | Karbwang, 1993(c) | Bradycardia developed in 9/29 patients with acute falciparum malaria; 1/6 healthy participants controls |
| Halofantrine, mefloquine | Nosten, 1993 | 1/61 developed bradycardia following halofantrine; 4/53 in mefloquine comparator group |
| Halofantrine | Lavallée, 2001 | 9-year-old girl developed ventricular extrasystoles with intermittent bradycardia, with an initially prolonged QTc of 480 ms which normalised to 420 ms after 48 hours (1/25 treated with halofantrine; no comparator group) |
| **Tachycardia** | | |
| Lumefantrine | Abdulla, 2008 | 7 cases of arrhythmia (mostly tachycardia): mild, resolved without intervention (4/447 in dispersible tablet group; 3/452 crushed tablet group) |
| **Ectopic beats** | | |
| Piperaquine | Karunajeewa, 2003 | 1 child developed frequent atrial ectopic beats at 24 hours, 1 adult developed ventricular ectopic beats 24 hours (1/62 patients) |
| **Supraventricular tachyarrhythmia** | | |
| Quinine, chloroquine | Bethell, 1996 | 8-year-old Gambian boy had frequent supraventricular ectopic beats but remained clinically undetectable, with 7 other patients experiencing SVTs (drug group not detailed; total trial population n=53). 2/23 patients treated with quinine had pauses: 17-year-old male, 1 pause of 2.3 s, 1 5-year-old female, 2 pauses 2.7 s and 2.4 s, and 1 patient had a couplet rhythm (2 ventricular beats in succession) |
| Quinine | Walker, 1993 | 2/29 patients (who died) developed SVT which authors felt was due to co-morbid state of patient; 0/25 in artemether group developed arrhythmias |
| Piperaquine, mefloquine | Gargano, 2012 | 20-year-old male WPW diagnosed on day 2 (previously undiagnosed), investigators thought this was possibly related to dihydroartemisinin-piperaquine (1/101 participants in piperaquine group; 0/49 developed arrhythmias in mefloquine group) |
| **Unspecified** | | |
| Quinine or mefloquine | Win, 1992 | 6 of 32 fatal cases (autopsy concluded death from cerebral malaria in all cases) developed one or more complications including cardiac dysrhythmias before dying (no further detail noted, treatment groups of fatal cases not detailed, total trial population n=141) |
| Lumefantrine | Kayentao, 2012 | 1/180 developed arrhythmia following lumefantrine thought possibly related to study drug; 0/355 following pyronaridine-artesunate |
| SP, sulfadoxine-pyrimethamine; ms, miliseconds; s, seconds; IM, intramuscular; SVT, supraventricular tachycardia; WPW, Wolff-Parkinson-White; QTc, corrected QT interval | | |
