# Supplementary material for: The arrhythmogenic cardiotoxicity of the quinoline and structurally related antimalarial drugs: a systematic review
Source: BMC Med. 2018 Nov 7;16:200. doi: 10.1186/s12916-018-1188-2 (PMC6220451; doi:10.1186/s12916-018-1188-2)
Supplement: Supplementary file 8 — Summary of secondary outcomes, for each drug. (DOCX 17 kb) [file 12916_2018_1188_MOESM8_ESM.docx]

| **Additional file 8** Summary of secondary outcomes, for each drug | | | | | | | | | |
| --- | --- | --- | --- | --- | --- | --- | --- | --- | --- |
|  | **Quinine (51 studies)** | **Mefloquine (45 studies)** | **Lumefantrine (39 studies)** | **Piperaquine (24 studies)** | **Halofantrine (22 studies)** | **Chloroquine (17 studies)** | **SP (14 studies)** | **Amodiaquine (10 studies)** | **Primaquine (7 studies)** |
| **Mean number of ECGs recorded per trial** | 7.6 | 8 | 6 | 4.3 | 8.2 | 7.6 | 5.6 | 5.8 | 8 |
| **Median number of ECGs recorded per trial** | 5.5 | 4 | 3 | 3 | 6 | 6 | 6 | 6 | 7 |
| **Number of trials using continuous ECG recording** | 5 | 1 | 2 | 1 | 2 | 3 | 0 | 0 | 0 |
| **% (number) trials unspecified method of reading QT** | 75 (38) | 78 (35) | 51 (20) | 54 (13) | 32 (7) | 71 (12) | 86 (12) | 60 (6) | 100 (7) |
| **% (number) trials manual method of reading QT** | 14 (7) | 9 (4) | 26 (10) | 33 (8) | 36 (8) | 12 (2) | 0 (0) | 30 (3) | 0 (0) |
| **% (number) trials automatic method of reading QT** | 4 (2) | 2 (1) | 3 (1) | 4 (1) | 5 (1) | 6 (1) | 0 (0) | 0 (0) | 0 (0) |
| **% (number) of trials automatic and manual method of reading QT** | 8 (4) | 11 (5) | 21 (8) | 8 (2) | 27 (6) | 12 (2) | 14 (2) | 10 (1) | 0 (0) |
| **% (number) trials unspecified method QT correction** | 67 (34) | 80 (36) | 33 (13) | 33 (8) | 22 (5) | 47 (8) | 79 (11) | 50 (5) | 57 (4) |
| **% (number) trials using Bazett's correction** | 31 (16) | 13 (6) | 28 (11) | 21 (5) | 77 (17) | 29 (5) | 21 (3) | 10 (1) | 0 (0) |
| **% (number) trials using Fridericia's correction** | 4 (2) | 2 (1) | 15 (6) | 21 (5) | 0 (0) | 18 (3) | 0 (0) | 10 (1) | 43 (3) |
| **% (number) trials using Bazett's and Fridericia's correction** | 0 (0) | 4 (2) | 23 (9) | 25 (6) | 0 (0) | 6 (1) | 0 (0) | 20 (2) | 0 (0) |
| **% (number) trials not specifying speed recording** | 71 (36) | 82 (37) | 82 (32) | 88 (21) | 64 (14) | 71 (12) | 93 (13) | 80 (8) | 100 (7) |
| **% (number) trials recording at 50mm/s** | 29 (15) | 16 (7) | 13 (5) | 0 (0) | 14 (3) | 12 (2) | 0 (0) | 20 (2) | 0 (0) |
| **% (number) trials which specify lead QT read from** | 20 (10) | 9 (4) | 10 (4) | 21 (5) | 27 (6) | 29 (5) | 14 (2) | 10 (1) | 0 (0) |
| **% (number) trials which specify food intake** | 12 (6) | 27 (12) | 69 (27) | 46 (11) | 64 (14) | 47 (8) | 21 (3) | 50 (5) | 86 (6) |
| **% (number) trials using ICH definition QTc prolongation** | 6 (3) | 11 (5) | 41 (16) | 48 (10) | 5 (1) | 18 (3) | 0 (0) | 20 (2) | 29 (2) |
| **% (number) trials not specifying a definition of prolongation** | 71 (36) | 80 (36) | 41 (16) | 50 (12) | 32 (7) | 54 (10) | 71 (10) | 60 (6) | 71 (5) |
| **% (number) trials which include PK data** | 37 (19) | 42 (19) | 49 (19) | 50 (12) | 46 (10) | 59 (10) | 36 (5) | 80 (8) | 57 (4) |
| **Median number PK samples taken in trials which collected PK** | 9.5 | 17 | 13 | 18.5 | 11.5 | 19.5 | 3 | 29 | 21 |

ICH, International Conference on Harmonisation of Technical Requirements for Registration of Pharmaceuticals for Human Use; PK, pharmacokinetic; SP, sulfadoxine-pyrimethamine.
