# Supplementary material for: The arrhythmogenic cardiotoxicity of the quinoline and structurally related antimalarial drugs: a systematic review
Source: BMC Med. 2018 Nov 7;16:200. doi: 10.1186/s12916-018-1188-2 (PMC6220451; doi:10.1186/s12916-018-1188-2)
Supplement: Supplementary file 9 — List of definitions of prolongation used per drug. (DOCX 18 kb) [file 12916_2018_1188_MOESM9_ESM.docx]

| **Additional file 9** List of definitions of prolongation used per drug | |  |
| --- | --- | --- |
| **Definition** | **Number of times definition used** | |
| **Quinine** |  | |
| ≥420 ms | 1 | |
| >420 ms (male), >430 ms (female) | 1 | |
| >440 ms | 4 | |
| >440 ms (Fridericia’s correction) | 1 | |
| >450 ms | 1 | |
| >500 ms | 1 | |
| >550 ms | 1 | |
| ≥570 ms | 1 | |
| Change from baseline >60 ms | 1 | |
| >5% of the baseline value | 1 | |
| >20% baseline value | 1 | |
| >25% of baseline | 6 | |
| **Mefloquine** |  | |
| ≥430 ms (male), ≥450 ms (female) | 1 | |
| >430 ms | 1 | |
| >440 ms | 2 | |
| >450 ms | 1 | |
| >30 ms with the value >450 ms (male), >470 ms (female) | 1 | |
| Borderline: 430 - 450 ms (adult males and children), 450 – 470 ms (adult females) | 1 | |
| Prolonged: > 450 ms (adult males and children), > 470 ms (adult females) | 1 | |
| >500 ms | 2 | |
| ≥550 ms | 1 | |
| >25% increase from baseline | 2 | |
| >30ms from baseline | 2 | |
| >60ms increase from baseline | 3 | |
| **Lumefantrine** |  | |
| >430 ms | 1 | |
| Borderline: 431 – 450 ms | 1 | |
| >30 ms with the value >430 ms (male), >450 ms (female) | 4 | |
| >440 ms | 2 | |
| >440 ms (Fridericia’s correction) | 1 | |
| ≥450 ms | 3 | |
| >450 ms (male), >470 ms (female) | 3 | |
| >460 ms | 1 | |
| >500 ms | 2 | |
| >500 ms (Fridericia’s correction) | 2 | |
| >25% increase from baseline | 2 | |
| >30 ms from baseline (Fridericia’s correction) | 1 | |
| >60 ms | 6 | |
| >60 ms from baseline (Fridericia’s correction) | 3 | |
| **Piperaquine** |  | |
| Borderline: 431 – 450 ms | 1 | |
| Borderline: 430 - 450 ms (adult males and children), 450 – 470 ms (adult females) | 2 | |
| ≥440 ms | 2 | |
| >450 ms | 4 | |
| >500 ms (Fridericia’s correction) | 4 | |
| Increase in >25% | 2 | |
| >30 ms | 2 | |
| >60 ms from baseline | 4 | |
| >60 ms from baseline (Fridericia’s correction) | 3 | |
| **Halofantrine** |  | |
| >420 ms | 1 | |
| >425 ms | 1 | |
| >30 ms with the value >430 ms (males), >450 ms (female) | 1 | |
| ≥440 ms | 8 | |
| ≥550 ms | 2 | |
| >25% from baseline | 6 | |
| **Chloroquine** |  | |
| >30 ms with the value >430 ms (male), >450 ms (female) | 1 | |
| >440 ms | 3 | |
| >450 ms | 2 | |
| >25% from baseline | 2 | |
| >30 ms from baseline (Fridericia’s correction) | 1 | |
| >60 ms from baseline | 1 | |
| **Sulfadoxine-pyrimethamine** |  | |
| >440 ms | 3 | |
| **Amodiaquine** |  | |
| >440 ms | 1 | |
| ≥450 ms | 1 | |
| >30 ms from baseline (Fridericia’s correction) | 1 | |
| >60 ms from baseline | 1 | |
| >60 ms from baseline (Fridericia’s correction) | 1 | |
| **Primaquine** |  | |
| ≥30 ms change from baseline | 1 | |
| ≥30 ms change from baseline (Fridericia’s correction) | 1 | |
| ≥30 ms change from baseline (Fridericia’s correction) | 1 | |
