# Supplementary material for: The arrhythmogenic cardiotoxicity of the quinoline and structurally related antimalarial drugs: a systematic review
Source: BMC Med. 2018 Nov 7;16:200. doi: 10.1186/s12916-018-1188-2 (PMC6220451; doi:10.1186/s12916-018-1188-2)
Supplement: Supplementary file 10 — Assessment of risk of bias for individual studies. (DOCX 26 kb) [file 12916_2018_1188_MOESM10_ESM.docx]

| **Additional file 10** Assessment of risk of bias for individual studies | | | | | | |
| --- | --- | --- | --- | --- | --- | --- |
| **Reference (first author, year of publication)** | **Blinding of participants and personnel** | **Blinding of outcome assessors** | **Incomplete outcome data** | **Selective outcome reporting** | **Number of ECGs** | **ECG methodology** |
| Roggelin, 2014 | Not blind, non-comparative trial | Not blind | Insufficient reporting of attrition/exclusions | No study protocol but outcomes reportedly planned in paper performed | 1 ECG performed in first 12 hours | 12 lead, 50mm/s, manually read |
| Humayun, 2013 | Not blind, non-comparative trial | Not blind | All participants accounted for | No study protocol | 2 ECGs in the first 12 hours | Speed of recording not outlined, 12 lead, method of ECG reading not outlined |
| Nyunt, 2012 | Not blind, non-comparative trial | Not blind | 1 patient excluded for adverse event, no justification for exclusion | No study protocol | 2 ECGs in first 12 hours | 12 lead, speed of recording not outlined, manually read |
| Khan, 2011 | Randomised, not blind | Not blind | Participants accounted for, no ECG data given | No study protocol | 1 ECG in first 12 hours | 12 lead, speed of recording not outlined, method of ECG reading not outlined |
| Rasheed, 2008 | Not blind, non-comparative trial | Not blind | Insufficient reporting of attrition/exclusions | No study protocol, no numerical data given | No ECG methodology detailed | No ECG methodology detailed |
| Noedl, 2006 | Randomised, not blind | Not blind | All randomised participants accounted for | No study protocol but outcomes reportedly planned in paper performed | 1 ECG performed in first 12 hours | 12 lead, speed of recording not outlined, method of ECG reading not outlined |
| Miller, 2006 | Randomised, not blind | Not blind | All randomised participants accounted for | No study protocol, no ECG planned analysis, no numerical data given | 1 ECG performed in first 12 hours | 12 lead, speed of recording not outlined, method of ECG reading not outlined |
| Haroon, 2005 | Randomised, not blind | Not blind | All randomised participants accounted for | No study protocol, no specific ECG planned analysis | 3 ECGs in first 12 hours | 12 lead, speed of recording not outlined, method of ECG reading not outlined |
| Bregani, 2004 | Randomised, not blind | Not blind | All participants accounted for | No study protocol, no specific ECG planned analysis | Ambulatory ECG for first 24 hours | Speed of recording not outlined, method of ECG reading not outlined |
| Assimadi, 2002 | Randomised, not blind | Not blind | All participants accounted for | No study protocol, no specific ECG planned analysis | 2 ECGs in first 12 hours | Speed of recording not outlined, method of ECG reading not outlined |
| Lefèvre, 2002(a) | Randomised, blind | Not blind | All participants accounted for | No study protocol but outcomes reportedly planned in paper performed | 25+ ECGs in first 12 hours | Speed of recording not outlined, 12 lead, manual and automatic reading of ECGs |
| Newton, 2001 | Randomised, not blind | Not blind | All participants accounted for | No study protocol but outcomes reportedly planned in paper performed | 2 ECGs in first 12 hours | Speed of recording not outlined, 12 lead, manual and automatic reading of ECGs |
| Thuma, 2000 | Randomised, not blind | Not blind | All participants accounted for | No study protocol, no ECG planned analysis | 1 ECG performed in first 12 hours | 12 lead, speed of recording not outlined, method of ECG reading not outlined |
| Claessen, 1998 | Case-control study | Not blind | All participants accounted for | No study protocol, no ECG planned analysis | Not detailed | 1 lead, speed not recorded, method of reading not outlined |
| Taylor, 1998 | Randomised, not blind | Not blind | Participants accounted for but no justification for exclusions | No study protocol, no ECG planned analysis, no numerical data given | 2 ECGs in first 12 hours | 12 lead, speed of recording not outlined, method of ECG reading not outlined |
| Phuong, 1997 | Randomised, not blind | Not blind | All participants accounted for | No study protocol, no ECG planned analysis, no numerical data given | Ambulatory ECG for first 24 hours | Speed of recording not outlined, method of ECG reading not outlined |
| Karbwang, 1997 | Randomised, not blind | Not blind | All participants accounted for | No study protocol but outcomes reportedly planned in paper performed | 2 ECGs in first 12 hours | 12 lead, speed of recording not outlined, manually and automatically read |
| Supanaranond, 1997 | Not blind, non-comparative trial | Not blind | All participants accounted for, appropriate exclusion | No study protocol but outcomes reportedly planned in paper performed | 16 ECGs in first 12 hours | 12 lead, 50mm/s, manually read |
| Hensbroek, 1996 | Non-randomised, non-blind comparative trial | Not blind | All participants accounted for | No study protocol but outcomes reportedly planned in paper performed | 3 ECGs in first 12 hours | 12 lead, 25 mm/sec + 50 mm/sec, manually read |
| Tran, 1996 | Randomised, blind | Not blind | All participants accounted for | No study protocol, no ECG planned analysis | 2 ECGs in first 12 hours | 1 lead, 50mm/s, method of reading not outlined |
| Murphy, 1996 | Randomised, not blind | Not blind | All participants accounted for, appropriate exclusions | No study protocol, no ECG planned analysis | 2 ECGs in first 12 hours | 12 lead, speed of recording not outlined, method of ECG reading not outlined |
| Bethell, 1996 | Non-randomised, non-blind comparative trial | Not blind | All participants accounted for | No study protocol but outcomes reportedly planned in paper performed | Ambulatory ECG for first 24 hours | Speed of recording not outlined, manual method of ECG reading |
| Sukontason, 1996 | Not blind, non-comparative trial | Not blind | All participants accounted for | No study protocol, no ECG planned analysis | 1 ECG performed in first 12 hours | 12 lead, speed of recording not outlined, method of ECG reading not outlined |
| Auprayoon, 1995 | Not blind, non-comparative trial | Not blind | All participants accounted for | No study protocol, no ECG planned analysis | Number of ECGs not detailed | 12 lead, speed of recording not outlined, method of ECG reading not outlined |
| Karbwang, 1995(a) | Randomised, not blind | Not blind | All participants accounted for, appropriate exclusions | No study protocol, no ECG planned analysis | 5 ECGs in first 12 hours | 12 lead, speed not recorded, automatic ECG reading |
| Watt, 1993 | Randomised, not blind | Blind | All participants accounted for, appropriate exclusions | No study protocol but outcomes reportedly planned in paper performed | 9 ECGs in first 12 hours | 12 lead, 50mm/s, method of reading ECGs not outlined |
| Karbwang, 1993(a) | Randomised, not blind | Not blind | All participants accounted for | No study protocol, no ECG planned analysis | 5 ECGs in first 12 hours | 12 lead, automatically read ECGs, speed not outlined |
| Walker, 1993 | Randomised, not blind | Not blind | All participants accounted for | No study protocol, no ECG planned analysis | 2 ECGs in first 12 hours | 12 lead, speed of recording not outlined, method of ECG reading not outlined |
| Karbwang, 1993(b) | Randomised, blind | Not blind | All participants accounted for | No study protocol, no ECG planned analysis | 6 ECGs in first 12 hours | 12 lead, 50mm/s, method of ECG reading not outlined |
| Supanaranond, 1993 | Non-randomised, non-blind comparative trial | Not blind | All participants accounted for | No study protocol but outcomes reportedly planned in paper performed | 16 ECGs in first 12 hours | 1 lead, 50mm/s, method of ECG reading not outlined |
| Win, 1992 | Randomised, blind | Not blind | All participants accounted for | No study protocol, no ECG planned analysis | 1 ECG performed in first 12 hours | 12 lead, speed of recording not outlined, method of ECG reading not outlined |
| Karbwang, 1992(a) | Randomised, not blind | Not blind | All participants accounted for | No study protocol, no ECG planned analysis | 2 ECGs in first 12 hours | 12 lead, speed of recording not outlined, method of ECG reading not outlined |
| Rai-Mra, 1991 | Non-randomised, blind | Not blind | All participants accounted for | No study protocol, no ECG planned analysis | 1 ECG performed in first 12 hours | 12 lead, 25 mm/sec + 50 mm/sec, manual method of ECG reading |
| Sowunmi, 1990 | Randomised, not blind | Not blind | All participants accounted for | No study protocol, no ECG planned analysis | 3 ECGs in first 12 hours | 12 lead, speed of recording not outlined, method of ECG reading not outlined |
| Mansor, 1990 | Not blind, non-comparative trial | Not blind | All participants accounted for | No study protocol, no ECG planned analysis | 3 ECGs in first 12 hours | 12 lead, 50mm/s, method of ECG reading not outlined |
| Davis, 1990 | Not blind, non-comparative trial | Not blind | All participants accounted for | No study protocol, no ECG planned analysis | 3 ECGs in first 12 hours | 12 lead, 50mm/s, method of ECG reading not outlined |
| Bunnag, 1989 | Randomised, blind | Not blind | All participants accounted for | No study protocol, no ECG planned analysis | 1 ECG performed in first 12 hours | 12 lead, speed of recording not outlined, method of ECG reading not outlined |
| Sabchareon, 1988 | Randomised, not blind | Not blind | No explanation for loss to follow up | No study protocol, no ECG planned analysis | 1 ECG performed in first 12 hours | 12 lead, speed of recording not outlined, method of ECG reading not outlined |
| Davis, 1988 | Not blind, non-comparative trial | Not blind | All participants accounted for | No study protocol, no ECG planned analysis | Ambulatory ECG for first 20 minutes | Ambulatory recorder, 50mm/s, method of ECG reading not outlined |
| Shwe, 1988 | Non-randomised, non-blind comparative trial | Not blind | All participants accounted for | No study protocol, no ECG planned analysis | Number of ECGs not detailed | 12 lead, speed of recording not outlined, method of ECG reading not outlined |
| Myint, 1987 | Non-randomised, non-blind comparative trial | Not blind | All participants accounted for | No study protocol, no ECG planned analysis | Number of ECGs not detailed | 12 lead, speed of recording not outlined, method of ECG reading not outlined |
| Wattanagoon, 1986 | Non-randomised, non-blind comparative trial | Not blind | All participants accounted for | No study protocol, no ECG planned analysis | 2 ECGs in first 12 hours | 12 lead, speed of recording not outlined, method of ECG reading not outlined |
| White, 1983(a) | Non-randomised, non-blind comparative trial | Not blind | All participants accounted for | No study protocol, no ECG planned analysis | 2 ECGs in first 12 hours | 12 lead, speed of recording not outlined, method of ECG reading not outlined |
| White, 1983(b) | Non-randomised, non-blind comparative trial | Not blind | All participants accounted for | No study protocol but outcomes reportedly planned in paper performed | 9 ECGs in first 12 hours | 12 lead, 50mm/s, method of ECG reading not outlined |
| White, 1983(c) | Non-randomised, non-blind comparative trial | Not blind | All participants accounted for | No study protocol, no ECG planned analysis | 10 ECGs in first 12 hours | 12 lead, 50mm/s, method of ECG reading not outlined |
| White, 1982 | Non-randomised, non-blind comparative trial | Not blind | All participants accounted for | No study protocol, no ECG planned analysis | 2 ECGs in first 12 hours | 12 lead, 25 mm/sec + 50 mm/sec, method of ECG reading not outlined |
| de Souza, 1983(a) | Randomised, blind | Not blind | All participants accounted for | No study protocol, no ECG planned analysis | Number of ECGs not detailed | 12 lead, speed of recording not outlined, method of ECG reading not outlined |
| de Souza, 1983(b) | Randomised, blind | Not blind | No explanation for loss to follow up | No study protocol, no ECG planned analysis | 1 ECG performed in first 12 hours | 12 lead, speed of recording not outlined, method of ECG reading not outlined |
| de Souza, 1985(a) | Randomised, blind | Not blind | No explanation for loss to follow up | No study protocol, no ECG planned analysis | 1 ECG performed in first 12 hours | 12 lead, speed of recording not outlined, method of ECG reading not outlined |
| de Souza, 1985(b) | Randomised, not blind | Not blind | All participants accounted for, appropriate exclusions | No study protocol, no ECG planned analysis | 1 ECG performed in first 12 hours | 12 lead, speed of recording not outlined, method of ECG reading not outlined |
| de Souza, 1987 | Randomised, blind | Not blind | All participants accounted for, appropriate exclusions | No study protocol, no ECG planned analysis | No ECGs in first 12 hours | 12 lead, speed of recording not outlined, method of ECG reading not outlined |
| Harinasuta, 1987 | Randomised, blind | Not blind | All participants accounted for, appropriate exclusions | No study protocol, no ECG planned analysis | 1 ECG performed in first 12 hours | 12 lead, speed of recording not outlined, method of ECG reading not outlined |
| Nosten, 1990 | Randomised, not blind | Not blind | All participants accounted for, appropriate exclusions | No study protocol, no ECG planned analysis | No ECGs in first 12 hours | 12 lead, speed of recording not outlined, method of ECG reading not outlined |
| Karbwang, 1991 | Randomised, blind | Not blind | All participants accounted for, appropriate exclusions | No study protocol, no ECG planned analysis | 13 ECGs in first 12 hours | 12 lead, speed of recording not outlined, method of ECG reading not outlined |
| Bunnag, 1992 | Randomised, not blind | Not blind | All participants accounted for, appropriate exclusions | No study protocol, no ECG planned analysis | Number of ECGs not detailed | 12 lead, speed of recording not outlined, method of ECG reading not outlined |
| Karbwang, 1992(b) | Randomised, not blind | Not blind | All participants accounted for, appropriate exclusions | No study protocol, no ECG planned analysis | 5 ECGs in first 12 hours | 12 lead, speed of recording not outlined, method of ECG reading not outlined |
| Karbwang, 1992(c) | Randomised, not blind | Not blind | All participants accounted for, appropriate exclusions | No study protocol, no ECG planned analysis | 5 ECGs in first 12 hours | 12 lead, speed of recording not outlined, method of ECG reading not outlined |
| Nosten, 1994(b) | Randomised, blind | Not blind | All participants accounted for, appropriate exclusions | No study protocol, no ECG planned analysis | 1 ECG performed in first 12 hours | 12 lead, speed of recording not outlined, method of ECG reading not outlined |
| Nosten, 1994(a) | Randomised, not blind | Not blind | All participants accounted for, appropriate exclusions | No study protocol, no ECG planned analysis | Number of ECGs not detailed | 12 lead, speed of recording not outlined, method of ECG reading not outlined |
| Karbwang, 1995(b) | Randomised, not blind | Not blind | All participants accounted for | No study protocol, no ECG planned analysis | 2 ECGs in first 12 hours | 12 lead, speed of recording not outlined, method of ECG reading not outlined |
| Karbwang, 1995(c) | Randomised, not blind | Not blind | All participants accounted for | No study protocol, no ECG planned analysis | 1 ECG performed in first 12 hours | 12 lead, speed of recording not outlined, method of ECG reading not outlined |
| ter Kuile, 1995 | Randomised, not blind | Not blind | All participants accounted for | No study protocol, no ECG planned analysis | 1 ECG performed in first 12 hours | 12 lead, speed of recording not outlined, method of ECG reading not outlined |
| Price, 1995 | Randomised, not blind | Not blind | All participants accounted for, appropriate exclusions | No study protocol, no ECG planned analysis | 1 ECG performed in first 12 hours | 12 lead, speed of recording not outlined, method of ECG reading not outlined |
| Jaspers, 1996 | Not blind, non-comparative trial | Not blind | All participants accounted for | No study protocol but outcomes reportedly planned in paper performed | No ECGs in first 12 hours | 12 lead, 25mm/s, method of ECG reading not outlined |
| Bangchang, 2000 | Not blind, non-comparative trial | Not blind | All participants accounted for | No study protocol, no ECG planned analysis | Number of ECGs not detailed | 12 lead, speed of recording not outlined, method of ECG reading not outlined |
| Massougbodji, 2002 | Randomised, blind | Not blind | All participants accounted for | No study protocol, no ECG planned analysis | 1 ECG performed in first 12 hours | 12 lead, speed of recording not outlined, method of ECG reading not outlined |
| Chanthap, 2005 | Not blind, non-comparative trial | Not blind | All participants accounted for | No study protocol, no ECG planned analysis | Number of ECGs not detailed | 12 lead, speed of recording not outlined, method of ECG reading not outlined |
| Bangchang, 2005 | Not blind, non-comparative trial | Not blind | All participants accounted for | No study protocol, no ECG planned analysis | No ECGs in first 12 hours | 12 lead, 25mm/s, method of ECG reading not outlined |
| Bhatt, 2006 | Not blind, non-comparative trial | Not blind | No explanation for loss to follow up | No study protocol, no ECG planned analysis | 1 ECG performed in first 12 hours | 12 lead, 25mm/s, method of ECG reading not outlined |
| Thuy, 2007 | Randomised, not blind | Not blind | All participants accounted for, appropriate exclusions | No study protocol, no ECG planned analysis | No ECGs in first 12 hours | 12 lead, 25mm/s, method of ECG reading not outlined |
| Pyar, 2007 | Randomised, blind | Not blind | All participants accounted for, appropriate exclusions | No study protocol, no ECG planned analysis | 1 ECG performed in first 12 hours | 12 lead, speed of recording not outlined, method of ECG reading not outlined |
| Bouyou-Akotet, 2010 | Randomised, not blind | Not blind | All participants accounted for, appropriate exclusions | Study protocol but no ECG planned analysis | 1 ECG performed in first 12 hours | 12 lead, speed of recording not outlined, method of ECG reading not outlined |
| Krudsood, 2010 | Randomised, not blind | Not blind | All participants accounted for, appropriate exclusions | No study protocol but outcomes reportedly planned in paper performed | 1 ECG performed in first 12 hours | 12 lead, speed of recording not outlined, method of ECG reading not outlined |
| Nasveld, 2010 | Randomised, blind | Not blind | All participants accounted for, appropriate exclusions | Study protocol but no ECG planned analysis | 1 ECG performed in first 12 hours | 12 lead, speed of recording not outlined, method of ECG reading not outlined |
| Krudsood, 2011 | Randomised, not blind | Not blind | All participants accounted for, appropriate exclusions | No study protocol but outcomes reportedly planned in paper performed | 1 ECG performed in first 12 hours | 12 lead, 50 mm/sec, manually interpreted ECGs |
| Rueangweerayut, 2012 | Randomised, not blind | Not blind | All participants accounted for, appropriate exclusions | Study protocol but no ECG planned analysis | 1 ECG performed in first 12 hours | 12 lead, speed of recording not outlined, method of ECG reading not outlined |
| Von Seidlein, 1997(b) | Not blind, non-comparative trial | Not blind | All participants accounted for, appropriate exclusions | No study protocol, no ECG planned analysis | 1 ECG performed in first 12 hours | 12 lead, speed of recording not outlined, method of ECG reading not outlined |
| Agtmael, 1999 | Randomised, blind | Not blind | All participants accounted for, appropriate exclusions | No study protocol but outcomes reportedly planned in paper performed | 1 ECG performed in first 12 hours | 12 lead, speed of recording not detailed, manually and automatically interpreted ECGs |
| Van Vugt, 1999(a) | Randomised, blind | Blind | All participants accounted for, appropriate exclusions | No study protocol, no ECG planned analysis | 1 ECG performed in first 12 hours | 12 lead, speed of recording not detailed, manually interpreted ECGs |
| Van Vugt, 1999(b) | Randomised, not blind | Not blind | No explanation for loss to follow up | No study protocol but outcomes reportedly planned in paper performed | 2 ECGs in first 12 hours | 12 lead, speed of recording not detailed, automatically interpreted ECGs |
| Van Vugt, 2000 | Randomised, not blind | Not blind | All participants accounted for, appropriate exclusions | No study protocol, no ECG planned analysis | 2 ECGs in first 12 hours | 12 lead, speed of recording not outlined, method of ECG reading not outlined |
| Bindschedler, 2000 | Randomised, blind | Blind | All participants accounted for, appropriate exclusions | No study protocol but outcomes reportedly planned in paper performed | 2 ECGs in first 12 hours | 12 lead, 50 mm/sec, automatically and manually interpreted ECGs |
| Ezzet, 2000 | Not randomised, blind | Not blind | All participants accounted for, appropriate exclusions | No study protocol, no ECG planned analysis | 1 ECG performed in first 12 hours | 12 lead, 50 mm/sec, automatically and manually interpreted ECGs |
| Kshirsagar, 2000 | Randomised, blind | Not blind | All participants accounted for, appropriate exclusions | No study protocol, no ECG planned analysis | 1 ECG performed in first 12 hours | 12 lead, speed of recording not outlined, manually interpreted ECGs |
| Lefevre, 2001 | Randomised, not blind | Blind | All participants accounted for, appropriate exclusions | No study protocol, no ECG planned analysis | 1 ECG performed in first 12 hours | 12 lead, 50 mm/sec, automatically and manually interpreted ECGs |
| Lefevre, 2002(b) | Randomised, not blind | Not blind | All participants accounted for, appropriate exclusions | No study protocol, no ECG planned analysis | 5 ECGs in first 12 hours | 12 lead, speed not outlined, automatically and manually interpreted ECGs |
| Bindschedler, 2002 | Randomised, blind | Blind | All participants accounted for, appropriate exclusions | No study protocol but outcomes reportedly planned in paper performed | 13 ECGs in first 12 hours | 12 lead, 50 mm/sec, automatically and manually interpreted ECGs |
| Falade, 2005 | Not blind, non-comparative trial | Blind | All participants accounted for, appropriate exclusions | No study protocol, no ECG planned analysis | 1 ECG performed in first 12 hours | 12 lead, speed of recording not detailed, manually interpreted ECGs |
| Alecrim, 2006 | Randomised, not blind | Not blind | All participants accounted for, appropriate exclusions | No study protocol, no ECG planned analysis | 1 ECG performed in first 12 hours | 12 lead, speed of recording not detailed, manually interpreted ECGs |
| McGready, 2006 | Not blind, non-comparative trial | Not blind | All participants accounted for, appropriate exclusions | No study protocol, no ECG planned analysis | 1 ECG performed in first 12 hours | 12 lead, speed of recording not outlined, method of ECG reading not outlined |
| Abdulla, 2008 | Randomised, blind | Not blind | All participants accounted for, appropriate exclusions | Study protocol but no ECG planned analysis | 1 ECG performed in first 12 hours | 12 lead, speed of recording not outlined, method of ECG reading not outlined |
| Falade, 2008 | Not blind, non-comparative trial | Blind | All participants accounted for, appropriate exclusions | Study protocol, outcomes planned performed | 1 ECG performed in first 12 hours | 12 lead, speed of recording not detailed, manually interpreted ECGs |
| Hatz, 2008 | Not blind, non-comparative trial | Blind | All participants accounted for, appropriate exclusions | No study protocol, no ECG planned analysis | 1 ECG performed in first 12 hours | 12 lead, 25mm/s, manually and automatically interpreted ECGs |
| McGready, 2008 | Randomised, not blind | Not blind | All participants accounted for, appropriate exclusions | Study protocol but no ECG planned analysis | 1 ECG performed in first 12 hours | 12 lead, speed of recording not outlined, method of ECG reading not outlined |
| Piola, 2010 | Randomised, not blind | Not blind | All participants accounted for, appropriate exclusions | Study protocol but no ECG planned analysis | 1 ECG performed in first 12 hours | 12 lead, speed of recording not outlined, method of ECG reading not outlined |
| Abdulla, 2010 | Randomised, not blind | Not blind | All participants accounted for, appropriate exclusions | No study protocol, no ECG planned analysis | 5 ECGs in first 12 hours | 12 lead, speed of recording not outlined, method of ECG reading not outlined |
| Tshefu, 2010 | Randomised, blind | Not blind | All participants accounted for, appropriate exclusions | Study protocol but no ECG planned analysis | 1 ECG performed in first 12 hours | 12 lead, speed of recording not detailed, manually interpreted ECGs |
| Ndiaye, 2011 | Randomised, not blind | Blind | All participants accounted for, appropriate exclusions | Study protocol but no ECG planned analysis | 1 ECG performed in first 12 hours | 12 lead, speed of recording not outlined, method of ECG reading not outlined |
| Kredo, 2011 | Randomised, not blind | Blind | All participants accounted for, appropriate exclusions | Study protocol, outcomes planned performed | 1 ECG performed in first 12 hours | 12 lead, speed of recording not detailed, manually interpreted ECGs |
| Byakika-Kibwika, 2011 | Not blind, non-comparative trial | Not blind | All participants accounted for, appropriate exclusions | Study protocol, outcomes planned performed | Ambulatory for first 12 hours | 12 lead ambulatory recorder, speed of recording not outlined, manually read ECGs |
| Adjei, 2012 | Randomised, blind | Not blind | All participants accounted for, appropriate exclusions | Study protocol, outcomes planned performed | 1 ECG performed in first 12 hours | 12 lead, 50 mm/sec, method of interpreting ECGs not outlined |
| Kayentao, 2012 | Randomised, not blind | Not blind | All participants accounted for, appropriate exclusions | Study protocol, outcomes planned performed | 1 ECG performed in first 12 hours | 12 lead, speed of recording not outlined, method of ECG reading not outlined |
| Kinde-Gazard, 2012 | Randomised, blind | Not blind | All participants accounted for, appropriate exclusions | No study protocol, no ECG planned analysis | 1 ECG performed in first 12 hours | 12 lead, speed of recording not outlined, method of ECG reading not outlined |
| Huang, 2012 | Not blind, non-comparative trial | Not blind | All participants accounted for, appropriate exclusions | Study protocol but no ECG planned analysis | 1 ECG performed in first 12 hours | 12 lead, speed of recording not outlined, method of ECG reading not outlined |
| Byakika-Kibwika, 2012 | Not blind, non-comparative trial | Not blind | All participants accounted for, appropriate exclusions | Study protocol, outcomes planned performed | Ambulatory for first 12 hours | 12 lead, speed of recording not outlined, method of ECG reading not outlined |
| Lefevre, 2013 | Randomised, not blind | Not blind | All participants accounted for, appropriate exclusions | Study protocol but no ECG planned analysis | No ECGs in first 12 hours | 12 lead, speed of recording not outlined, method of ECG reading not outlined |
| Kakuda, 2013 | Randomised, not blind | Not blind | No explanation of exclusions for adverse events | No study protocol, no ECG planned analysis | No ECGs in first 12 hours | 12 lead, speed of recording not outlined, method of ECG reading not outlined |
| Laman, 2014 | Randomised, not blind | Not blind | All participants accounted for, appropriate exclusions | Study protocol but no ECG planned analysis | 1 ECG performed in first 12 hours | 12 lead, speed of recording not outlined, method of ECG reading not outlined |
| Karunajeewa, 2003 | Not blind, non-comparative trial | Not blind | All participants accounted for, appropriate exclusions | No study protocol but outcomes reportedly planned in paper performed | 1 ECG performed in first 12 hours | 12 lead, 25mm/s, manual method of ECG reading |
| Ashley, 2004 | Randomised, blind | Not blind | All participants accounted for, appropriate exclusions | No study protocol, no ECG planned analysis | 2 ECGs in first 12 hours | 12 lead, speed of recording not detailed, automatically interpreted ECGs |
| Roshammar, 2006 | Not blind, non-comparative trial | Not blind | All participants accounted for, no loss to follow up | No study protocol, no ECG planned analysis | 1 ECG performed in first 12 hours | 12 lead, speed of recording not outlined, method of ECG reading not outlined |
| Mytton, 2007 | Randomised, not blind | Not blind | All participants accounted for | No study protocol but outcomes reportedly planned in paper performed | 2 ECGs in first 12 hours | 12 lead, 25mm/s, manual method of ECG reading |
| Trung, 2009 | Randomised, not blind | Not blind | No explanation of loss to follow up | No study protocol, no ECG planned analysis | 1 ECG performed in first 12 hours | 12 lead, speed of recording not outlined, method of ECG reading not outlined |
| Pyar, 2009 | Randomised, not blind | Not blind | All participants accounted for | No study protocol, no ECG planned analysis | 1 ECG performed in first 12 hours | 12 lead, speed of recording not outlined, method of ECG reading not outlined |
| Bassat, 2009 | Randomised, not blind | Blind | All participants accounted for | Study protocol, outcomes planned performed | 1 ECG performed in first 12 hours | 12 lead, speed of recording not specified, manual method of ECG reading |
| Valecha, 2010 | Randomised, not blind | Not blind | All participants accounted for | Study protocol, outcomes planned performed | 1 ECG performed in first 12 hours | 12 lead, speed of recording not specified, manual method of ECG reading |
| Mayxay, 2010 | Randomised, not blind | Not blind | All participants accounted for | Study protocol, outcomes planned performed | 1 ECG performed in first 12 hours | 12 lead, speed of recording not specified, manual method of ECG reading |
| Song, 2011 | Randomised, not blind | Not blind | No explanation of reasons why loss to follow up | Study protocol but no ECG planned analysis | 1 ECG performed in first 12 hours | 12 lead, speed of recording not outlined, method of ECG reading not outlined |
| Hien, 2011 | Randomised, not blind | Not blind | All participants accounted for | No study protocol, no ECG planned analysis | 1 ECG performed in first 12 hours | 12 lead, speed of recording not outlined, method of ECG reading not outlined |
| Tjitra, 2012 | Randomised, not blind | Not blind | All participants accounted for | No study protocol, no ECG planned analysis | 4 ECGs in first 12 hours | 12 lead, speed of recording not outlined, method of ECG reading not outlined |
| Valecha, 2012 | Randomised, not blind | Not blind | All participants accounted for | Study protocol but no ECG planned analysis | 3 ECGs in first 12 hours | 12 lead, speed of recording not outlined, method of ECG reading not outlined |
| Gargano, 2012 | Randomised, not blind | Not blind | All participants accounted for | Study protocol but no ECG planned analysis | 1 ECG performed in first 12 hours | 12 lead, speed of recording not outlined, method of ECG reading not outlined |
| Lon, 2014 | Randomised, not blind | Not blind | All participants accounted for | Study protocol but no ECG planned analysis | Number of ECGs not detailed | 12 lead, speed of recording not specified, manual method of ECG reading |
| Moore, 2014 | Randomised, not blind | Not blind | All participants accounted for | No study protocol, no ECG planned analysis | 2 ECGs in first 12 hours | 12 lead, speed of recording not specified, manual method of ECG reading |
| Manning, 2014 | Randomised, blind | Not blind | All participants accounted for, appropriate exclusions | Study protocol, outcomes planned performed | 2 ECGs in first 12 hours | 12 lead, 25mm/s, manual and automatic method of ECG reading |
| Bigira, 2014 | Randomised, not blind | Not blind | All participants accounted for, appropriate exclusions | Study protocol but no ECG planned analysis | No ECGs in first 12 hours | 12 lead, speed of recording not outlined, method of ECG reading not outlined |
| Hanboonkunupakarn, 2014 | Randomised, not blind | Not blind | All participants accounted for | Study protocol but no ECG planned analysis | 6 ECGs in first 12 hours | 12 lead, speed of recording not outlined, method of ECG reading not outlined |
| Baiden, 2015 | Not blind, non-comparative trial | Not blind | All participants accounted for, appropriate exclusions | Study protocol, outcomes planned performed | 1 ECG performed in first 12 hours | 12 lead, speed of recording not specified, manual and automatic method of ECG reading |
| Benjamin, 2015 | Randomised, not blind | Not blind | All participants accounted for, appropriate exclusions | No study protocol, no ECG planned analysis | 3 ECGs in first 12 hours | 12 lead, speed of recording not outlined, method of ECG reading not outlined |
| Darpo, 2015 | Randomised, blind | Not blind | All participants accounted for, appropriate exclusions | Study protocol, outcomes planned performed | 6 ECGs in first 12 hours | 12 lead, speed of recording not outlined, method of ECG reading not outlined |
| Stein, 2015 | Randomised, not blind | Not blind | All participants accounted for, appropriate exclusions | No study protocol but outcomes reportedly planned in paper performed | 5 ECGs in first 12 hours | 12 lead, speed of recording not specified, manual method of ECG reading |
| Toure, 2016 | Randomised, blind | Not blind | All participants accounted for, appropriate exclusions | Study protocol but no ECG planned analysis | 1 ECG performed in first 12 hours | 12 lead, speed of recording not outlined, method of ECG reading not outlined |
| Krishna, 1993 | Not blind, non-comparative trial | Not blind | All participants accounted for | No study protocol, no ECG planned analysis | 4 ECGs in first 12 hours | 12 lead, 25mm/s, manual and automatic method of ECG reading |
| Nosten, 1993 | Non-randomised, not blind | Blind | All participants accounted for | No study protocol, no ECG planned analysis | 4 ECGs in first 12 hours | 12 lead, 50 mm/s, manual and automatic method of ECG reading |
| Karbwang, 1993(c) | Not blind, non-comparative trial | Not blind | All participants accounted for | No study protocol, no ECG planned analysis | 5 ECGs in first 12 hours | 12 lead, speed of recording not detailed, manual method of ECG reading |
| Monlun, 1995 | Not blind, non-comparative trial | Not blind | All participants accounted for | No study protocol but outcomes reportedly planned in paper performed | 2 ECGs in first 12 hours + ambulatory for first 48 hours | 12 lead, speed of recording not detailed, manual method of ECG reading |
| Matson, 1996 | Not blind, non-comparative trial | Blind | All participants accounted for | No study protocol but outcomes reportedly planned in paper performed | 1 ECG performed in first 12 hours | 12 lead, speed of recording not detailed, manual method of ECG reading |
| Touze, 1996 | Not blind, non-comparative trial | Not blind | All participants accounted for | No study protocol but outcomes reportedly planned in paper performed | 3 ECGs in first 12 hours + ambulatory for first 48 hours | Ambulatory recorder, 25 mm/s, method of ECG reading not outlined |
| Restepo, 1996 | Randomised, blind | Not blind | All participants accounted for | No study protocol, no ECG planned analysis | 1 ECG performed in first 12 hours | 12 lead, speed of recording not specified, manual method of ECG reading |
| Hombhanje, 1998 | Not blind, non-comparative trial | Not blind | No explanation of why patient did not complete follow up | No study protocol, no ECG planned analysis | 3 ECGs in first 12 hours + ambulatory for first 48 hours | 12 lead, 25mm/s, manual method of ECG reading |
| Sowumni, 1998 | Not blind, non-comparative trial | Not blind | All participants accounted for | No study protocol but outcomes reportedly planned in paper performed | 2 ECGs in first 12 hours | 12 lead, 25mm/s, manual and automatic method of ECG reading |
| Lavallée, 2001 | Not blind, non-comparative trial | Not blind | All participants accounted for | No study protocol, no ECG planned analysis | 1 ECG performed in first 12 hours | 12 lead, speed of recording not detailed, automatically interpreted ECGs |
| Touze, 2002 | Randomised, not blind | Not blind | All participants accounted for, appropriate exclusions | No study protocol but outcomes reportedly planned in paper performed | Ambulatory for first 24 hours in addition to 2 12 lead ECGs | Ambulatory and 12 lead, 25 and 50mm/s, automatic and manual method of reading |
| Minodier, 2005 | Non-randomised, not blind | Not blind | All participants accounted for, appropriate exclusions | No study protocol, no ECG planned analysis | 1 ECG performed in first 12 hours | 12 lead, speed of recording not outlined, method of ECG reading not outlined |
| Khan, 2006 | Not blind, non-comparative trial | Not blind | All participants accounted for | No study protocol, no ECG planned analysis | 1 ECG performed in first 12 hours | 12 lead, speed of recording not detailed, manual method of ECG reading |
| Omoruyi, 2007 | Randomised, not blind | Not blind | All participants accounted for | No study protocol, no ECG planned analysis | 3 ECGs in first 12 hours + ambulatory for first 48 hours | 12 lead, speed of recording not detailed, manual method of ECG reading |
| Siriez, 2012 | Retrospective study | Not blind | All participants accounted for | No study protocol, no ECG planned analysis | 1 ECG performed in first 12 hours | 12 lead, speed of recording not detailed, manual method of ECG reading |
| Bouchaud, 2000 | Randomised, not blind | Not blind | All participants accounted for, appropriate exclusions | No study protocol, no ECG planned analysis | 3 ECGs in first 12 hours + ambulatory for first 48 hours | 12 lead, speed of recording not outlined, method of ECG reading not outlined |
| Khan, 2005 | Not blind, non-comparative trial | Not blind | All participants accounted for, appropriate exclusions | No study protocol, no ECG planned analysis | Not detailed | 12 lead, speed of recording not outlined, method of ECG reading not outlined |
| Babalola, 2009 | Randomised, not blind | Not blind | All participants accounted for, appropriate exclusions | No study protocol, no ECG planned analysis | Not detailed | 12 lead, speed of recording not outlined, method of ECG reading not outlined |
| Bassi, 2004 | Randomised, not blind | Not blind | All participants accounted for, appropriate exclusions | No study protocol, no ECG planned analysis | 4 ECGs in first 12 hours + ambulatory for first 48 hours | 12 lead, speed of recording not outlined, method of ECG reading not outlined |
| Pukrittayakamee, 2014 | Randomised, not blind | Not blind | All participants accounted for, appropriate exclusions | Study protocol, outcomes planned performed | 6 ECGs in first 12 hours | 12 lead, speed of recording not outlined, method of ECG reading not outlined |
| Llanos-Cuentas, 2014 | Randomised, blind | Not blind | All participants accounted for, appropriate exclusions | Study protocol, outcomes planned performed | 2 ECGs in first 12 hours | 12 lead, speed of recording not outlined, method of ECG reading not outlined |
| Miller, 2013 | Randomised, blind | Blind | All participants accounted for, appropriate exclusions | Study protocol, outcomes planned performed | 3 ECGs in first 12 hours + ambulatory for first 48 hours | 12 lead ambulatory, speed of recording not outlined, method of ECG reading not outlined |
| Oluwafunmilayo, 2011 | Non-randomised, non-blind control trial | Not blind | All participants accounted for, appropriate exclusions | No study protocol, no ECG planned analysis | 2 ECGs in first 12 hours | 12 lead, 25mm/s, automatically interpreted ECGs |
| Poravuth, 2011 | Randomised, blind | Not blind | All participants accounted for, appropriate exclusions | Study protocol, outcomes planned performed | 1 ECG performed in first 12 hours | 12 lead, speed of recording not outlined, method of ECG reading not outlined |
| Latha, 2010 | Randomised, blind | Not blind | All participants accounted for | No study protocol, no ECG planned analysis | 2 ECGs in first 12 hours | 12 lead, speed of recording not outlined, method of ECG reading not outlined |
| Mzayek, 2007 | Randomised, blind | Not blind | All participants accounted for | Study protocol, outcomes planned performed | Ambulatory recording after dosing in addition to 12 lead ECGs | Ambulatory and 12 lead, speed not outlined, automatic and manual method of reading |
| Cook, 2006 | Randomised, not blind | Not blind | All participants accounted for, appropriate exclusions | No study protocol, no ECG planned analysis | 2 ECGs in first 12 hours | 12 lead, speed of recording not outlined, method of ECG reading not outlined |
| Sowunmi, 1999 | Randomised, not blind | Not blind | All participants accounted for | No study protocol but outcomes reportedly planned in paper performed | 2 ECGs in first 12 hours | 12 lead, 25mm/s, method of ECG reading not outlined |
| Von Seidlein, 1997(a) | Randomised, not blind | Not blind | All participants accounted for | No study protocol but outcomes reportedly planned in paper performed | 1 ECG performed in first 12 hours | 12 lead, 25mm/s, manual and automatic method of ECG reading |
| Bangchang, 1994 | Randomised, not blind | Not blind | All participants accounted for | No study protocol, no ECG planned analysis | 1 ECG performed in first 12 hours | 12 lead, speed of recording not outlined, method of ECG reading not outlined |
| White, 1988 | Randomised, not blind | Not blind | All participants accounted for | No study protocol, no ECG planned analysis | 10 ECGs in first 12 hours | 3 lead ECG, 50mm/s, method of ECG reading not outlined |
| Edwards, 1988 | Randomised, not blind | Not blind | All participants accounted for | No study protocol, no ECG planned analysis | 1 ECG performed in first 12 hours | 12 lead, speed of recording not outlined, method of ECG reading not outlined |
| Looareesuwan, 1986 | Randomised, not blind | Not blind | All participants accounted for | No study protocol, no ECG planned analysis | 11 ECGs in first 12 hours | 12 lead, 50mm/s, manual method of ECG reading |
| Gustafsson, 1983 | Randomised, not blind | Not blind | All participants accounted for | No study protocol, no ECG planned analysis | Not detailed | 12 lead, speed of recording not outlined, method of ECG reading not outlined |
| Euke, 1987 | Randomised, not blind | Not blind | All participants accounted for, appropriate exclusions | No study protocol, no ECG planned analysis | 1 ECG performed in first 12 hours | 12 lead, speed of recording not outlined, method of ECG reading not outlined |
| Thapa, 2007 | Randomised, not blind | Not blind | All participants accounted for, appropriate exclusions | Study protocol, outcomes planned performed | 1 ECG performed in first 12 hours | 12 lead, speed of recording not outlined, method of ECG reading not outlined |
| Mutabingwa, 2009 | Randomised, not blind | Not blind | All participants accounted for, appropriate exclusions | Study protocol but no ECG planned analysis | 1 ECG performed in first 12 hours | 12 lead, speed of recording not outlined, method of ECG reading not outlined |
| White, 1987 | Not blind, non-comparative trial | Not blind | All participants accounted for | No study protocol, no ECG planned analysis | 8 ECGs in first 12 hours | Rhythm strip ECG, 50mm/s, method of ECG reading not outlined |
| Ngouesse, 2001 | Randomised, not blind | Not blind | All participants accounted for, appropriate exclusions | No study protocol but outcomes reportedly planned in paper performed | 2 ECGs in first 12 hours | 12 lead, speed of recording not outlined, manual and automatic method of ECG reading |
| Orrell, 2008 | Randomised, not blind | Not blind | All participants accounted for, appropriate exclusions | No study protocol, no ECG planned analysis | 2 ECGs in first 12 hours | 12 lead, speed of recording not outlined, manual method of ECG reading |
| Navaratnam, 2009 | Randomised, not blind | Not blind | All participants accounted for, appropriate exclusions | No study protocol, no ECG planned analysis | 4 ECGs in first 12 hours | 12 lead, speed of recording not outlined, method of ECG reading not outlined |
| Ogutu, 2014 | Randomised, not blind | Not blind | All participants accounted for, appropriate exclusions | No study protocol but outcomes reportedly planned in paper performed | 3 ECGs in first 12 hours | 12 lead, speed of recording not outlined, manual method of ECG reading |
| Liu, 2014 | Randomised, not blind | Not blind | All participants accounted for, appropriate exclusions | No study protocol, no ECG planned analysis | Not detailed | 12 lead, speed of recording not outlined, method of ECG reading not outlined |
| Gogtay, 2006 | Randomised, not blind | Not blind | No explanation of why patients did not complete follow up | No study protocol, no ECG planned analysis | 1 ECG performed in first 12 hours | 12 lead, speed of recording not outlined, method of ECG reading not outlined |
| Jittamala, 2015 | Randomised, not blind | Not blind | All participants accounted for, appropriate exclusions | Study protocol, outcomes planned performed | 6 ECGs in first 12 hours | 12 lead, speed of recording not outlined, method of ECG reading not outlined |
