# Supplementary material for: The arrhythmogenic cardiotoxicity of the quinoline and structurally related antimalarial drugs: a systematic review
Source: BMC Med. 2018 Nov 7;16:200. doi: 10.1186/s12916-018-1188-2 (PMC6220451; doi:10.1186/s12916-018-1188-2)

Additional file 11 Risk of bias for each criterion per drug

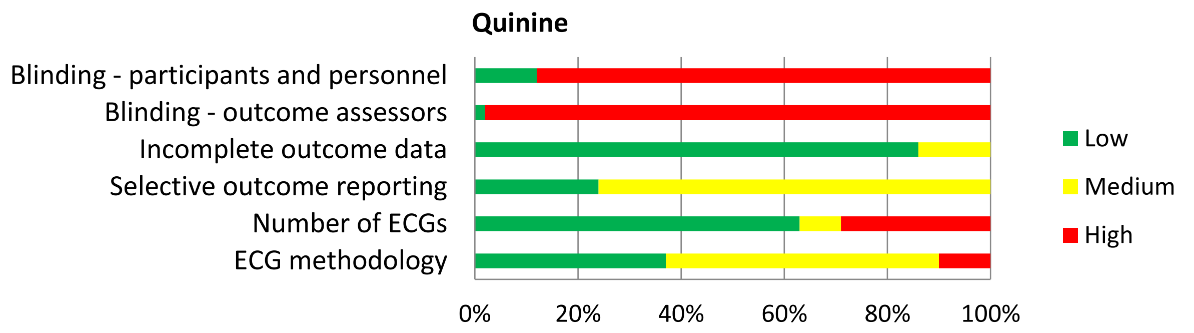

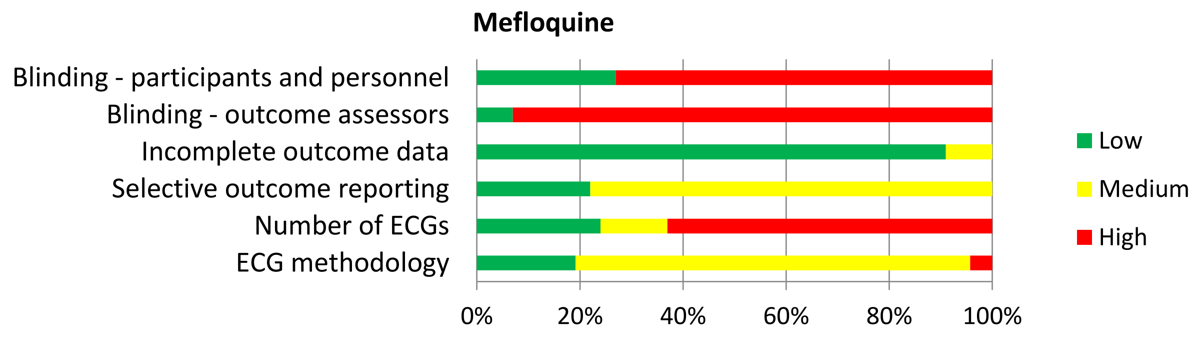

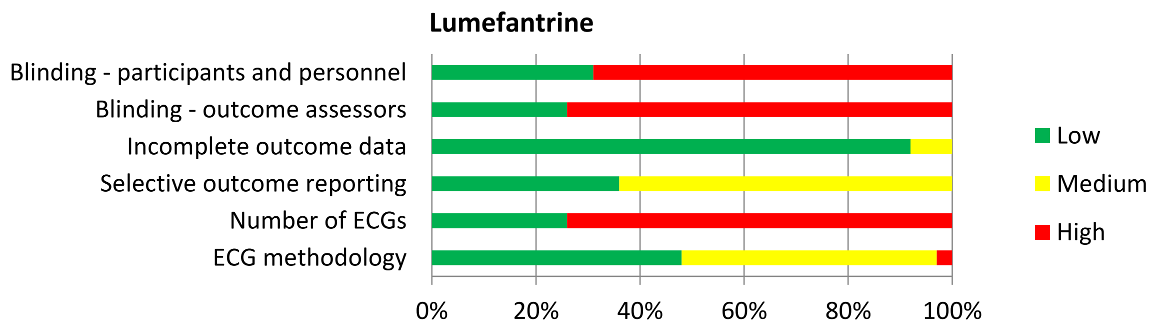

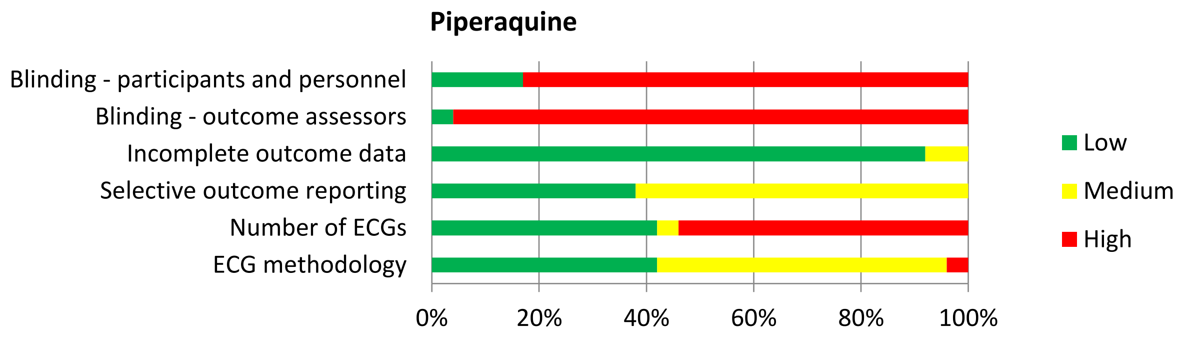
Piperaquine
Blinding - participants and personnel
Blinding - outcome assessors
Incomplete outcome data
Selective outcome reporting
Number of ECGs
ECG methodology
0%
20%
40%
60%
80%
100%
Low
Medium
High

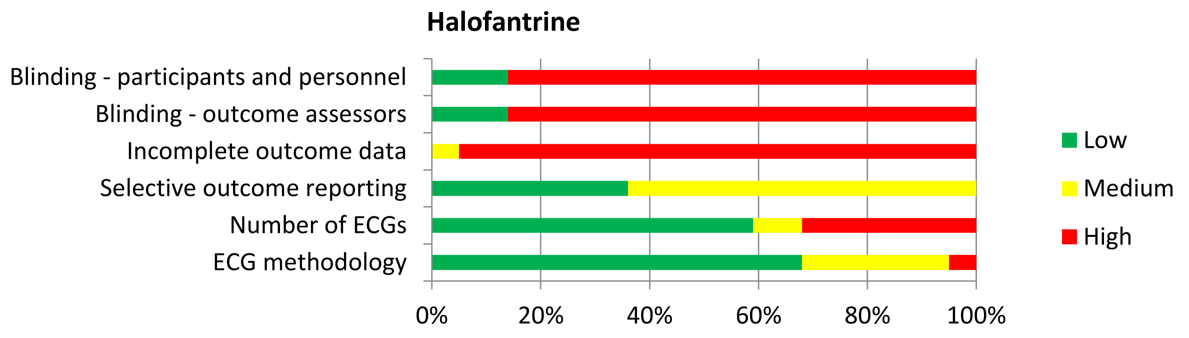
Halofantrine
Blinding - participants and personnel
Blinding - outcome assessors
Incomplete outcome data
Selective outcome reporting
Number of ECGs
ECG methodology
0%
20%
40%
60%
80%
100%
Low
Medium
High

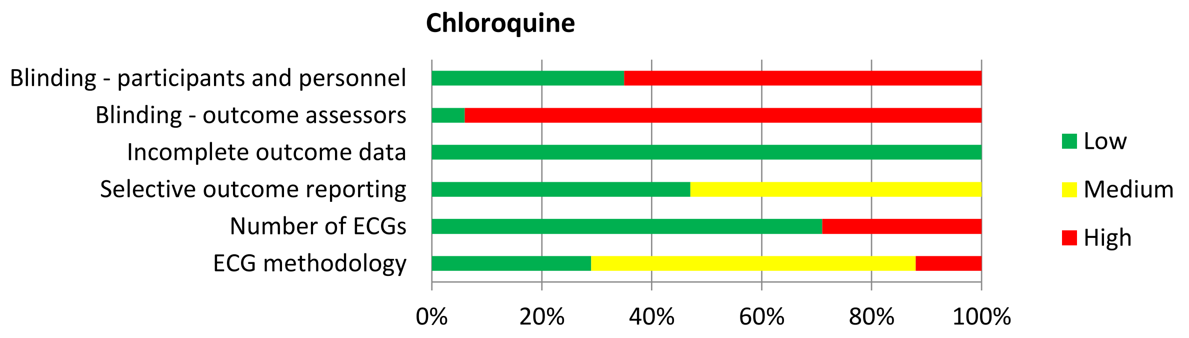
Chloroquine
Blinding - participants and personnel
Blinding - outcome assessors
Incomplete outcome data
Selective outcome reporting
Number of ECGs
ECG methodology
0%
20%
40%
60%
80%
100%
Low
Medium
High

Sulfadoxine-pyrimethamine

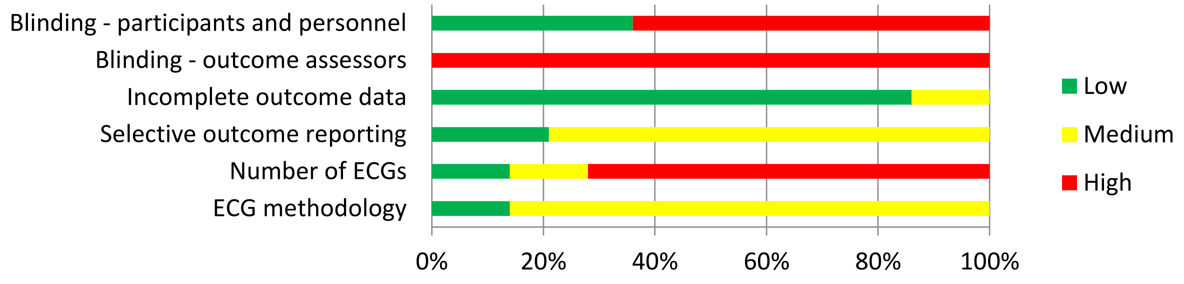

Amodiaquine

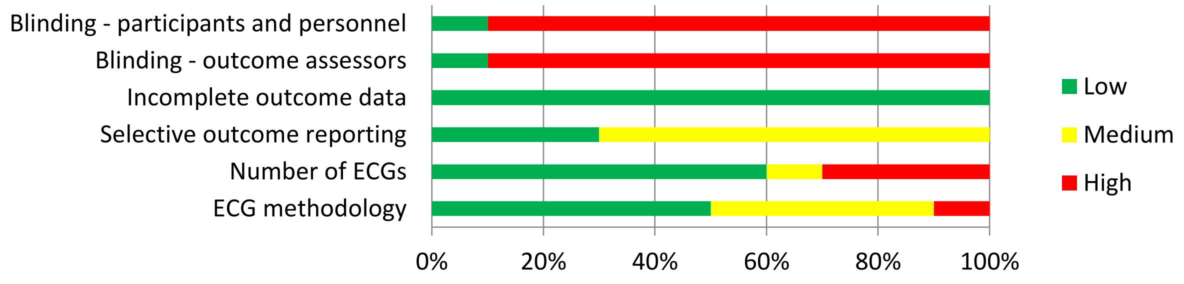

Primaquine

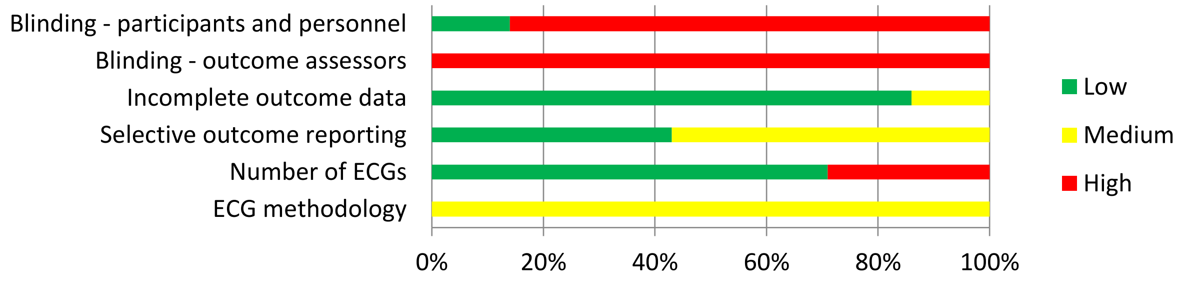

Supplement: Supplementary file 11 — Risk of bias for each criterion per drug. (PDF 621 kb) [file 12916_2018_1188_MOESM11_ESM.pdf]
